# Unified transient creep constitutive model based on the crack evolution of micritic bioclastic limestone

**DOI:** 10.1371/journal.pone.0276100

**Published:** 2022-10-27

**Authors:** Zuguo Mo, Li Qian, Tianzhi Yao, Yunpeng Gao, Fujun Xue, Jianhai Zhang, Ru Zhang, Enlong Liu

**Affiliations:** State Key Laboratory of Hydraulics and Mountain River Engineering, College of Water Resources and Hydropower, Sichuan University, Chengdu, China; University of Vigo, SPAIN

## Abstract

The surrounding rock at the exit of the No. 1 drainage tunnel of the Artashi Water Conservancy Project is micritic bioclastic limestone with 55% bioclastic material. This rock underwent unpredictable large and time-dependent deformation during excavation. To date, the mechanical behaviour of this kind of rock has rarely been studied. In this study, traditional triaxial compression tests and multilevel creep tests were conducted on micritic bioclastic limestone, and the results clarified the instantaneous and time-dependent mechanical properties of the rock. Considering that the essence of rock failure is crack growth, the crack strain evolution properties were revealed in rock triaxial compression tests and multilevel creep tests. Based on triaxial compression tests, the evolution of axial cracks with increasing deviatoric stress ratio *R_d_* (ratio of deviatoric stress to peak deviatoric stress) was observed, and an axial crack closure element and new crack growth element were proposed. To simulate the creep behaviour of a rock specimen, the relationship of the rock creep crack strain rate with *R_d_* was studied. A creep crack element was created, and the creep crack strain evolution equation was obtained, which closely fit the experimental data. Combining the 4 element types (elastic element, crack closure element, crack growth element, and creep crack element), a unified transient creep constitutive model (Mo’s model) was proposed, which represented both the transient and time-dependent mechanical properties of the micritic bioclastic limestone.

## 1. Introduction

**Fig 1:**
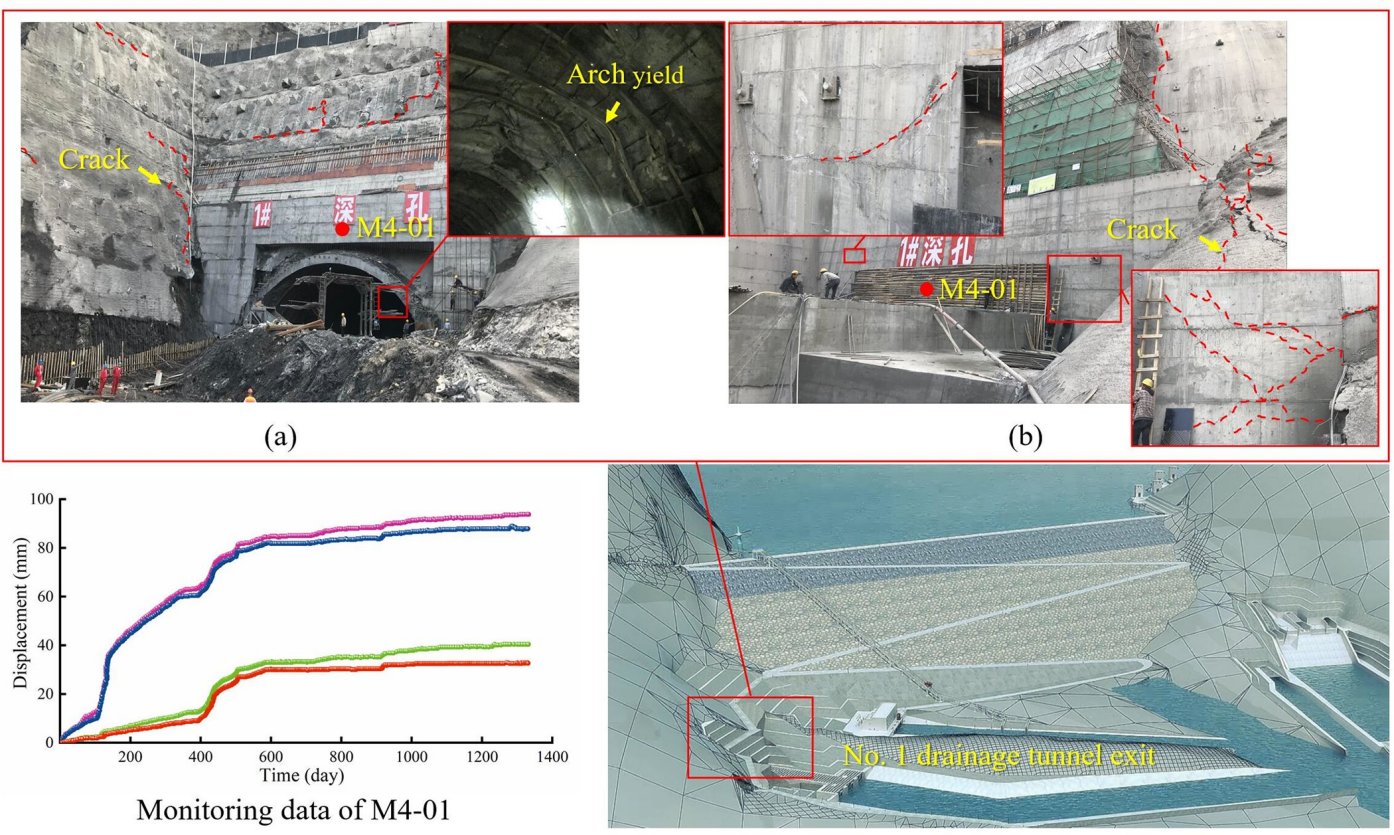
Deformation and safety problems of the No. 1 drainage tunnel: (a) initial support, (b) secondary support.

The Artashi Water Conservancy Project in Xinjiang Province, China, is the controlling project on the Yarkant River, undertaking flood control, irrigation diversion and power generation. The surrounding rock at the exit of No. 1 drainage tunnel of the Artashi Water Conservancy Project has experienced unexpected large and continuous deformation, leading to safety problems of the tunnel and slope. The deformation of the surrounding rock and slope have increased with time, resulting in fracture of the slope concrete and yield failure of the steel arch of the tunnel (Fig 1(A)). The relative displacement of the outer surface of the arch top recorded by a 4-point monitor (M4-01) was 93.77 mm after 1332 days. The slope and the tunnel exit were reinforced with anchor cable and concrete anchor supports. Then monitoring and on-site observations revealed that slope deformation continued and the support structure underwent cracking failure, as shown in Fig 1(B). Therefore, it is necessary to investigate the composition and mechanical properties of the rock to reveal the mechanisms of rock deformation.

**Fig 2:**
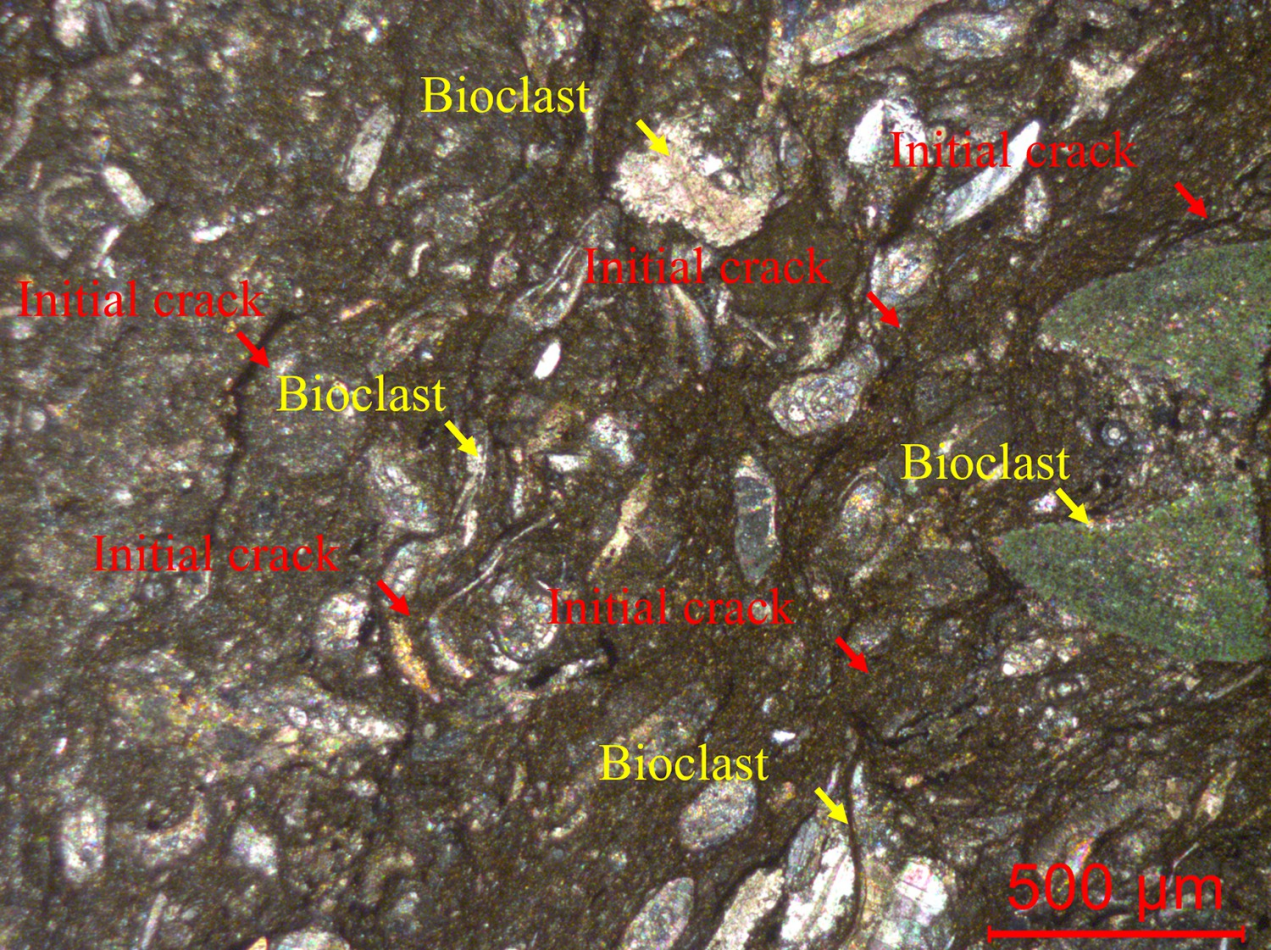
Micritic bioclastic limestone composition, as observed under a polarizing microscope.

The surrounding rock is black and has a relatively dense massive structure, and thin section analysis results show that the rock has a biological skeleton structure (Fig 2). Numerous incipient cracks can also be seen in the rock. The composition of this rock is 45% interstitial material and 55% bioclastic material, occasionally with terrigenous clastic material. The widely distributed interstitial material is mainly a micrite substrate composed of micrite calcite. Bioclasts are mostly round, oval, tubular, curved and irregular in shape, and the grains vary in size, with diameters mostly less than 2 mm. The particle diameter size of the terrigenous clastic material is generally smaller than 0.05 mm, mainly quartz, and sporadically distributed. Accordingly, this rock is named micritic bioclastic limestone.

To the authors’ knowledge, there are very few reports of in-depth studies of this kind of rock. This type of rock was previously encountered in engineering, mainly near faults or weak structural surfaces, and rarely with bioclastic limestone as the tunnel surrounding rock. Studies on limestone containing bioclastic material have been conducted by many scholars. EL-Sorogy AS et al. [1] performed a study on the composition and diagenesis of reefal limestone in central Saudi Arabia. Rožič B et al. [2] studied the composition of Middle Jurassic limestone megabreccia from the Slovenian Basin and inferred the evolution of the platform margin. V. Vajdova et al. [3, 4] used microscopy to study microstructural damage and pore changes in porous carbonates containing bioclasts after triaxial compression experiments at different confining pressures; the results revealed that the microscopic damage mechanism was pore collapse and crack coalescence. Ji. Y et al. [5] used X-ray microtomography 3D imaging to study the pore changes in Indiana limestone containing bioclastic material after compression experiments and observed the pore reduction characteristics. The mechanical properties of reef limestone, which also contained bioclasts, have been progressively studied in recent years. Chang-qi Z et al. [6] investigated the microstructures and uniaxial compressive strength of beach calcarenites in the South China Sea. Q S. Meng et al. [7] conducted Hopkinson pressure bar tests to study the dynamic failure properties of coral reef limestones. The crack initiation and damage evolution of micritized framework reef limestone from the South China Sea were studied by triaxial compression tests [8]. However, research on the time-dependent mechanical properties of micritic bioclastic limestone has rarely been reported. As mentioned by H. Liu et al. [8], the mechanical properties of reef limestone depend on their composition, structure and diagenesis. The complex composition and specific structure of this micritic bioclastic limestone make it very unique, and thus, it is valuable to study the mechanical properties of this rock.

The phenomenon of time-dependent deformation of the No. 1 drainage tunnel surrounding rock and slope is a typical creep-type deformation [9–11]. Many scholars have conducted numerous creep tests, such as single-stage, multilevel creep tests and loading cycle creep tests, on different rocks to investigate their creep behaviour [9, 11–15]. To characterize the creep evolution of rocks, empirical models, component models, and other novel models have been proposed. An empirical model can represent the creep properties of a particular rock accurately with relatively few parameters. The drawback of an empirical model is that the parameters always lack physical meaning. Empirical models are only suitable for describing creep test data at specific stresses and is difficult to apply them to make engineering predictions for complex stress conditions [16–19]. For a component model, a spring, dashpot and slider are used to represent the elastic, viscous and plastic mechanical response of rock, respectively. With the combination of elements, this model can represent the primary and secondary creep stages, such as the models of Maxwell, Kelvin, Burgers, and Nishihara [20–24]. Fractional order has been introduced into the viscous element to represent the tertiary creep stage [25–29]. A component model is relatively easy to understand, and the components and parameters have physical meaning. However, a component model is phenomenologically proposed based on the mechanical response of rock and not based on the mechanism of creep [11, 30].

To study the creep properties of rocks in depth and to develop constitutive models, important work has been performed by a number of scholars in recent years. A novel damage-based creep model has been proposed based on the time-hardening and damage theory of rock [30]. Zhang Xing et al. [31] established the damage evolution equation based on the variation in the deformation modulus of rock in multistage creep experiments, and then the creep equation was established. Based on the growth model of a single crack, scholars have used the micro/macro approach to establish the relationship between crack extension and macrostrain and proposed the brittle creep model [32]. Yanlin Zhao et al. [33] Based on wing crack propagation theory, crack propagation properties were investigated, and an equivalent Burgers model was proposed to characterise the normalised crack propagation length. With the development of particle flow codes, numerical methods have been adopted to investigate the creep properties of rocks. Hua Li et al. [34] proposed a 3D microcreep model to simulate the creep properties of rock salt based on PFC3D, which agreed well with indoor tests. Kefeng Zhou et al. [35] investigated the effect of shear parameters on the creep properties of rocks containing joints based on PFC numerical software. The microscopic mechanism of rock creep and damage has been generally accepted as the evolution of microcracks. The crack strain proposed by Martin [36] ignored the complex and random distribution of initial cracks and thus avoided the difficulties caused by uncertainty [37]. In the present study, micritic bioclastic limestone was obtained from the Artashi Water Conservancy Project, and conventional triaxial and multilevel loading creep tests were conducted. The crack strain characteristics of rock specimens in triaxial compression and creep tests were revealed, and the crack evolution at all stages of the creep process was investigated. A unified transient creep constitutive model based on crack evolution was proposed.

## 2. Triaxial compression and creep tests

### 2.1 Rock experiment preparation and procedures

**Fig 3:**
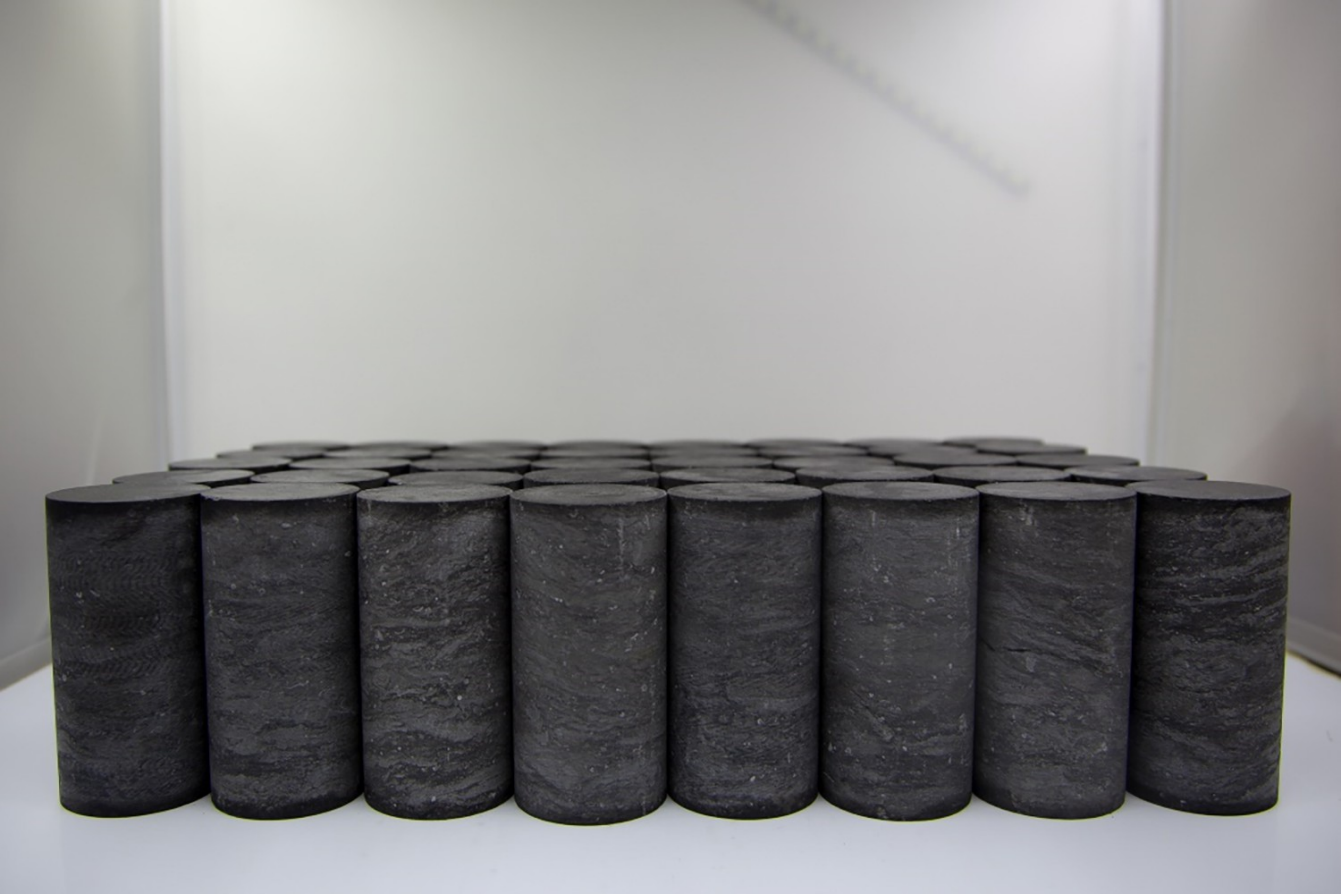
Rock specimens.

The rock used to make the specimens was taken from the Artashi Water Conservancy Project in Xinjiang, China. A whole rock block, approximately 900 kg, was obtained from the surrounding rock of the large-deformation tunnel to reduce variability among the rock specimens. All rock samples were drilled in the same direction and carefully processed into standard cylindrical rock specimens with dimensions of Φ50 mm×100 mm (Fig 3) and a height-to-diameter ratio of 2.0. The average density of the rock specimens is 2.77 g/cm^3^.

The confining pressures adopted in the conventional triaxial compression testing were 1, 3, 5 and 10 MPa, with 5 specimens tested under each condition. The loading equipment in these tests was the MTS815 apparatus at Sichuan University. The specifications of the loading equipment are shown in Table 1 [38].

**Table 1:** Specifications of MTS815.

Maximum axial force	Maximum confining pressure	Operating temperature	Extensometer resolution		Measured accuracy
			Axial	Circumferential	
4600 kN	140 MPa	20~200°C	±4 mm	-2.5~8 mm	0.5%

The conventional triaxial compression tests of the micritic bioclastic limestone were performed as follows:

(1) The confining pressure was applied to a predetermined value with a loading rate of 6 MPa/min.

(2) An axial load was applied at a loading rate of 20 kN/min.

(3) The axial load control method was changed to lateral deformation rate control with 0.02 mm/min at the end of the elastic stage of the rock specimen.

(4) After the peak stress, the axial load was applied at a lateral deformation rate of 0.04 mm/min until the residual deformation stage of the rock was reached.

The multilevel loading creep tests on the micritic bioclastic limestone were carried out in the following steps:

(1) The confining pressure and axial load were applied in the same way as in the conventional triaxial compression test.

(2) After the axial load was applied to a predetermined value, the deviatoric stress was kept constant for a predetermined time (2 h).

(3) After the creep period ended, the load was unloaded at a rate of 20 kN/min until the deviatoric stress reached 0.25 MPa, which was held for 0.2 h.

(4) The next deviatoric stress level was applied, and steps (2) and (3) were repeated until the specimen failed.

The confining pressure remained constant for each level of the multilevel loading creep tests. Based on the stress thresholds of the triaxial compression test, as a load level reference, 4 to 8 deviatoric stress levels were applied, such as *σ*_*ci*_, (*σ*_*ci*_+*σ*_*cd*_)/2, *σ*_*cd*_, 1.05*σ*_*cd*,_ 1.1*σ*_*cd*_, 1.15*σ*_*cd*_, 1.20*σ*_*cd*_, and 1.25*σ*_*cd*_. To ensure that the rock was not broken during the loading process, the rock loading stress was adjusted according to the rock strain response. The confining pressures of the triaxial compression and multilevel loading creep tests are set to 1 MPa, 3 MPa, 5 MPa and 10 MPa.

### 2.2 Laboratory test of micritic bioclastic limestone

**Fig 4:**
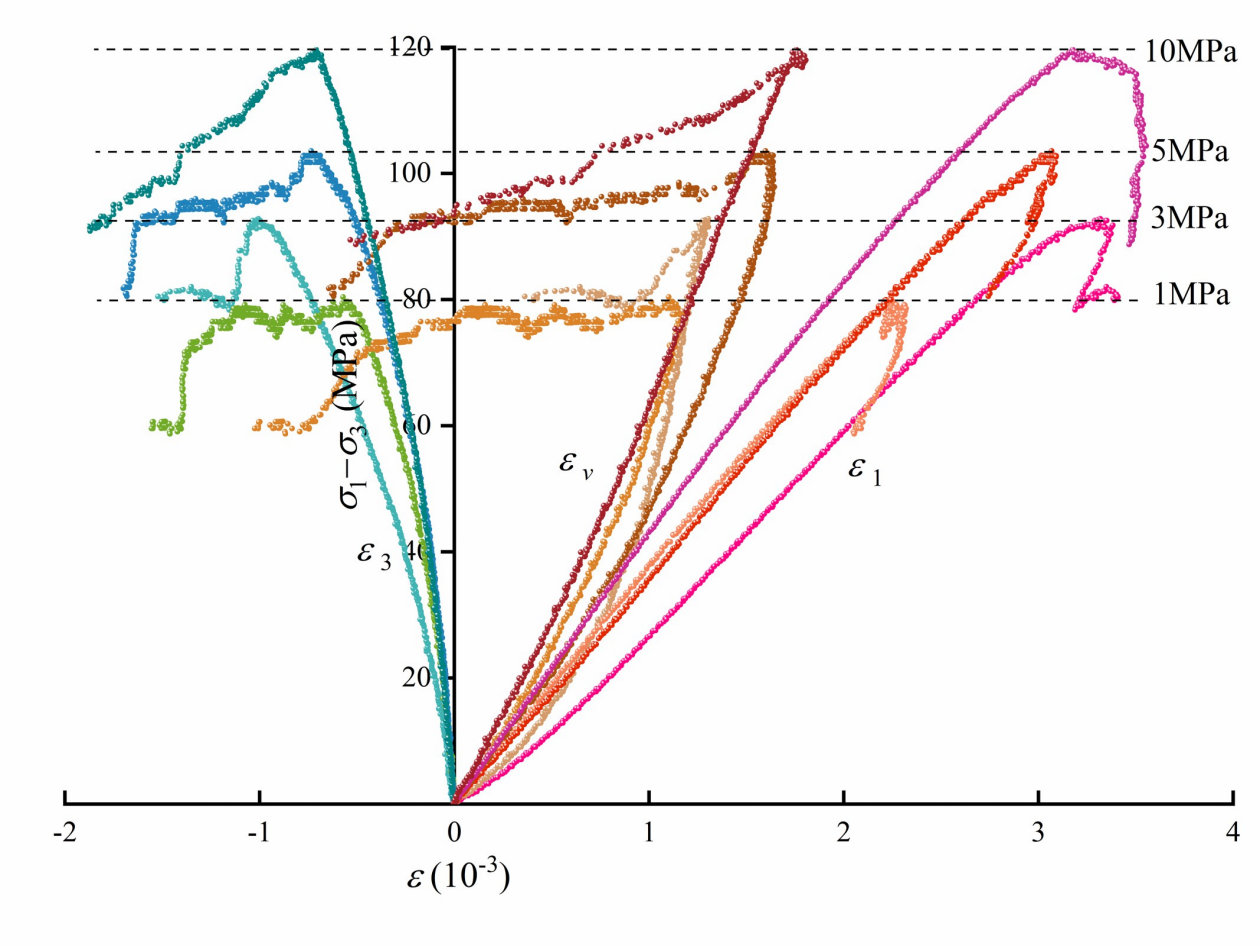
Triaxial compression test results of micritic bioclastic limestone.

To study the time-dependent deformation and crack evolution characteristics of the tunnels, conventional triaxial and multilevel loading creep test experiments on the rock were carried out. The results of the triaxial compression tests are shown in Fig 4. The peak deviatoric stresses of the micritic bioclastic limestone are 79.10 MPa, 92.81 MPa, 104.77 MPa and 137.04 MPa at confining pressures of 1 MPa, 3 MPa, 5 MPa and 10 MPa, respectively, showing a positive linear relationship with confining pressure. The elastic moduli of the micritic bioclastic limestone are 38.05 GPa, 32.89 GPa, 37.76 GPa and 45.83 GPa, classifying it as a moderately hard rock.

Based on the results of the triaxial compression tests, the stress thresholds of micritic bioclastic limestone are determined in Section 3. Considering the rock strain response during the test, the adjusted deviatoric stress of multilevel loading creep tests are shown in Table 2.

**Table 2:** Deviatoric stress at each level.

Confining pressure	Deviatoric stress							
	Level 1	Level 2	Level 3	Level 4	Level 5	Level 6	Level 7	Level 8
1 MPa	47.05	53.18	63.58	74.35	83.93	90.96	-	-
3 MPa	48.90	66.16	79.29	91.79	-	-	-	-
5 MPa	56.90	68.26	81.75	95.14	102.20	108.64	115.87	121.59
10 MPa	77.81	91.48	109.75	119.16	127.75	136.95	-	-

**Fig 5:**
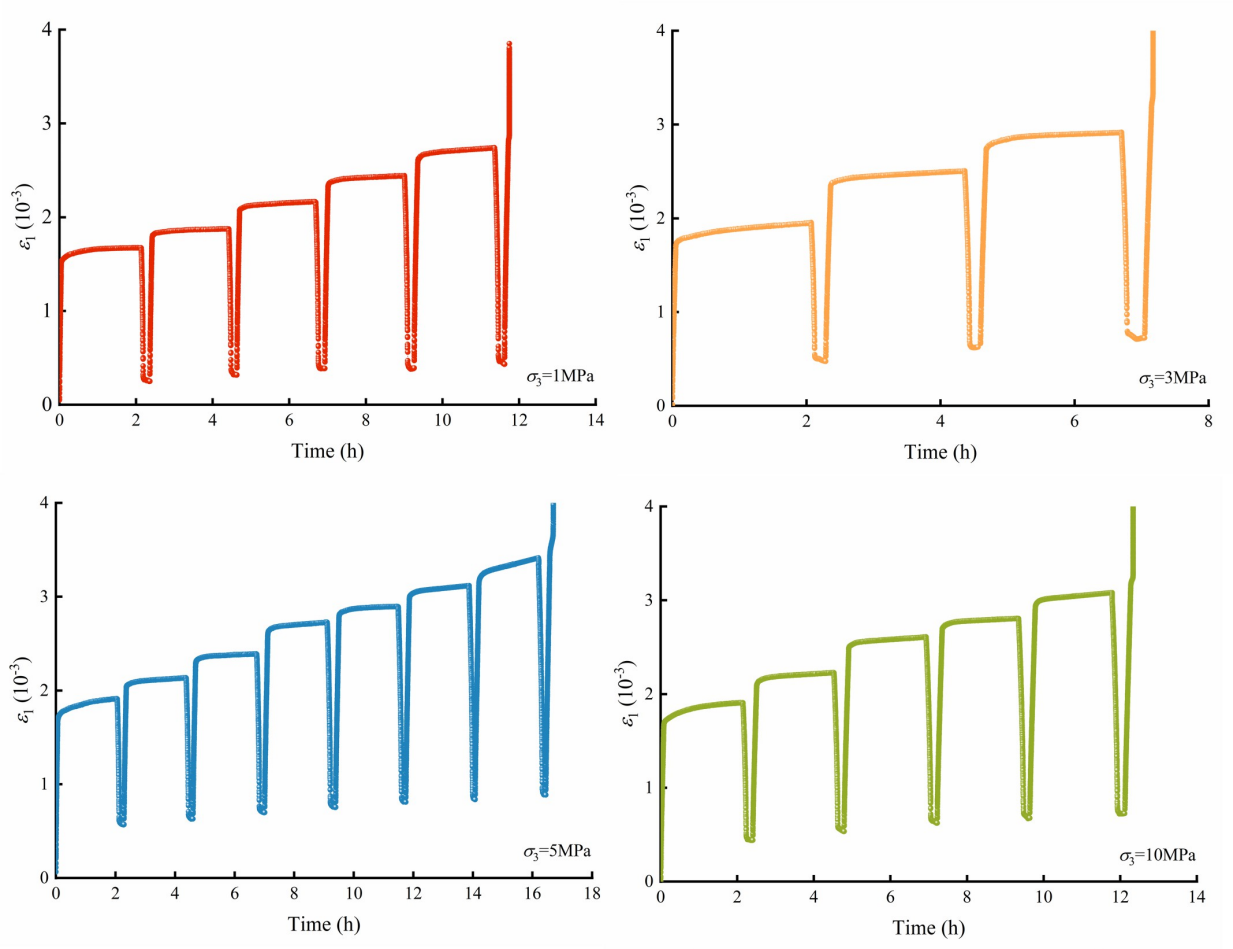
Crack strain evolution characteristics.

The multilevel loading rock creep tests were carried out by MTS815, and the results are shown in Fig 5. As the load reaches the predetermined value, the axial strain of the rock evolves with time, exhibiting significant creep characteristics. The axial strain and the slope of the curves increase with the loading level.

## 3. Crack strain evolution characteristics

### 3.1 Crack strain calculation and stress threshold determination

Axial and lateral strains arise when rock specimens are subjected to forces, including elastic and crack strains. The elastic strain of loaded specimens in a conventional triaxial compression test can be calculated by Hooke’s law and can be denoted by *ε*_1*e*_ and *ε*_3*e*_ in the axial and lateral directions:

$$\left\{ \begin{array}{l} \varepsilon_{1e}^{}=\frac{1}{E}(\sigma_{1}-2\mu_{e}\sigma_{3})\\ \varepsilon_{3e}^{}=\frac{1}{E}[\sigma_{3}-\mu_{e}(\sigma_{1}+\sigma_{3})] \end{array}\right.$$ (1)

where *ε*_1*e*_ and *ε*_3*e*_ denote the axial and lateral elastic strains, respectively, *E* is the elastic modulus, *μ*_*e*_ is the elastic Poisson’s ratio, *σ* 1 denotes the axial stress, and *σ* 3 is the confining pressure.

From Eq (1), the axial strain increment independent of Poisson’s ratio can be expressed as:

$$\Delta \varepsilon_{1e}^{}=\varepsilon_{1e}^{1}-\varepsilon_{1e}^{0}=\frac{1}{E}\left(\sigma_{1}^{1}-\sigma_{1}^{0}\right)$$ (2)

According to Reference [39], crack strain refers to the strain resulting from the closure, propagation and coalescence of cracks. The crack strain of the rock specimens includes the axial crack strain *ε*_1*c*_ and the lateral crack strain *ε*_3*c*_. The axial and lateral strains obtained from triaxial compression tests consist of elastic and crack strains, which are presented in Eq (3):

$$\left\{ \begin{array}{l} \varepsilon_{1}=\varepsilon_{1e}^{}+\varepsilon_{1c}^{}\\ \varepsilon_{3}=\varepsilon_{3e}^{}+\varepsilon_{3c}^{} \end{array}\right.$$ (3)

where *ε*_1_ denotes the axial strain and *ε*_3_ denotes the lateral strain.

Therefore, the rock crack strain can be obtained from Eq (4):

$$\left\{ \begin{array}{l} \varepsilon_{1c}^{}=\varepsilon_{1}-\frac{1}{E}(\sigma_{1}-2\mu_{e}\sigma_{3})\\ \varepsilon_{3c}^{}=\varepsilon_{3}-\frac{1}{E}[\sigma_{3}-\mu_{e}(\sigma_{1}+\sigma_{3})] \end{array}\right.$$ (4)

**Fig 6:**
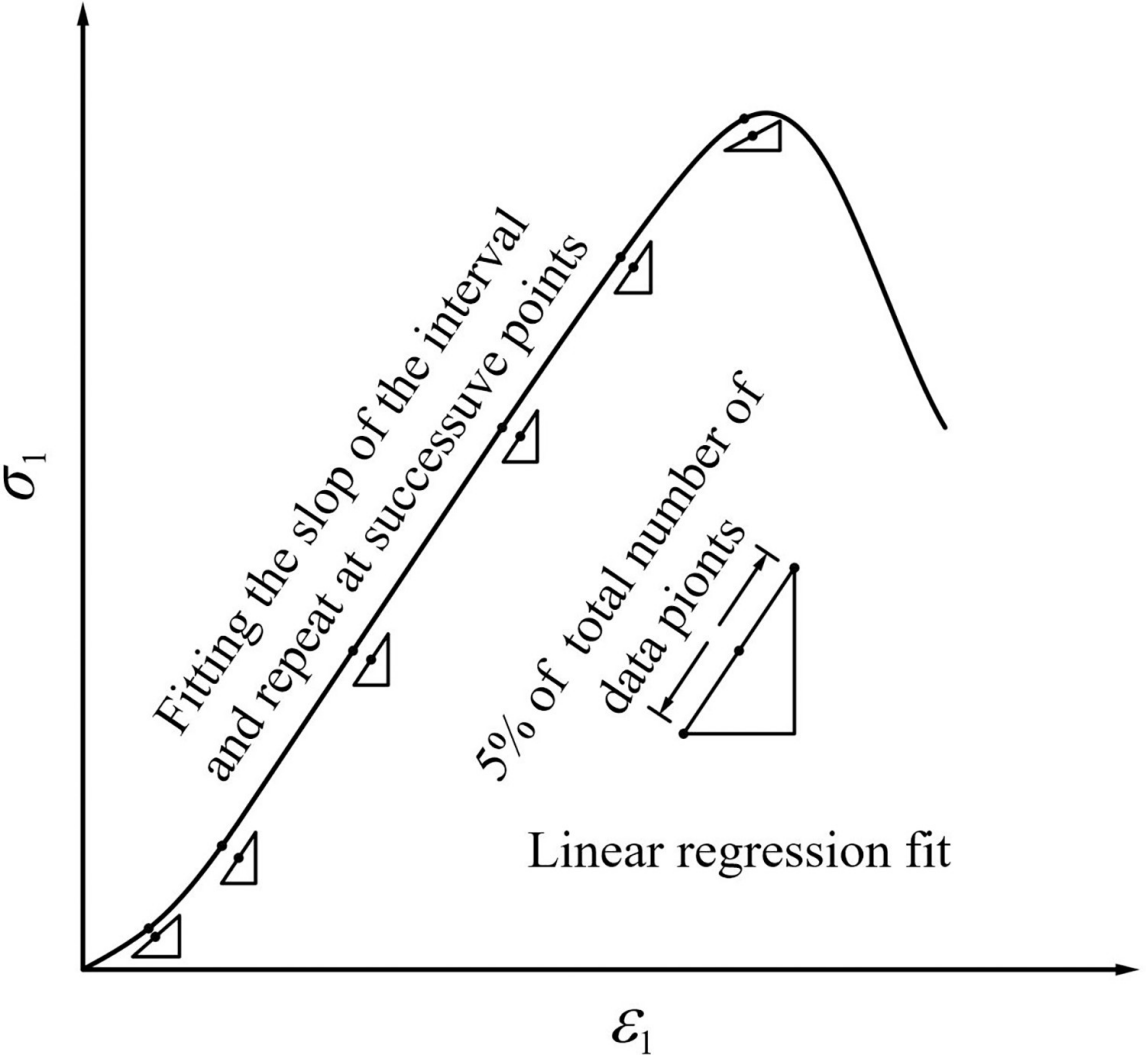
Moving point regression technique (after reference [43]).

The rock crack strain in Eq (4) is sensitive to the elastic parameters (*E* and *μ*_*e*_). Thus, *E* and *μ*_*e*_ of the rock specimen must be obtained accurately. The elastic modulus *E* of rock specimens under triaxial compression can be obtained by the slope of the linear portion of the axial stress–strain curve [40, 41]. A method called the moving point regression technique, which uses a sliding window approach to move the axial strain–stress dataset to fit a straight line over an interval, has been proposed to obtain the elastic modulus of rocks [42]. According to Reference [42], a relatively smooth tangent modulus curve can be obtained when 5% of the total number of data points is within a certain interval (Fig 6). Eq (1) indicates that the lateral elastic strain depends on the elastic Poisson’s ratio. To accurately determine the elastic Poisson’s ratio of rock, the moving point regression technique is adopted.

Reference [44] defined the failure process of brittle rock in five stages by four stress thresholds: crack closure stress *σ*_*cc*_, crack initiation stress *σ*_*ci*_, crack damage *σ*_*cd*_ stress and peak stress *σ*_*p*_. Reference [45] reported many methods for determining the stress thresholds of rock based on previous studies. However, the deviatoric stress–volumetric strain curve shown in Fig 4 of micritic bioclastic limestone under triaxial compression has no typical reversal point, so the method proposed in Reference [36, 45] cannot be used in determining *σ*_*cd*_. Therefore, an optimized method to determine the stress thresholds of rocks is proposed as follows.

1. Determine the elastic modulus (*E*) of the rock by the moving point regression technique.
2. Calculate the axial crack strain. The deviatoric stress–axial crack strain curve is plotted.
3. Find the beginning point of the vertical section of the deviatoric stress–axial crack strain curve, which can be determined as *σ*_*cc*_.
4. Calculate the instantaneous Poisson’s ratio by the moving point regression technique and determine the elastic Poisson’s ratio (*μ*_*e*_) at *σ*_*cc*_ determined in step (3).
5. Calculate the lateral crack strain and volumetric crack strain with the parameters obtained by steps (1) and (4).
6. Plot the deviatoric stress against the lateral and volumetric crack strains.
7. Determine *σ*_*ci*_ by the point at the end of the vertical section of the deviatoric stress-lateral crack strain curve.
8. Determine *σ*_*cd*_ as the point at the end of the vertical section of the deviatoric stress–axial crack strain curve.

In step (3), *σ*_*cc*_ is determined by the axial crack strain instead of the volumetric crack strain, thus avoiding the summation of errors and improving the accuracy of the results. In step (8), the axial crack strain curve is used instead of the volumetric strain curve, overcoming the problem caused by the absence of a typical reversal arc and reversal point of the volumetric strain curve. The stress thresholds obtained by Martin’s method [36] and the present method are listed in Table 3. The stress thresholds (*σ*_*cc*_ and *σ*_*ci*_) determined by the two methods are close, whereas *σ*_*cd*_ obtained by the present method is more reasonable than Martin’s method.

**Table 3:** Stress thresholds determined with different methods.

Stress threshold determination method	*σ*_*cc*_	*σ*_*ci*_	*σ*_*cd*_	*σ*_*p*_
Martin’s method	18.36	32.73	92.81	92.81
Present method	26.82	31.81	65.07	92.81

### 3.2 Crack evolution characteristics of triaxial compression tests and creep tests

**Fig 7:**
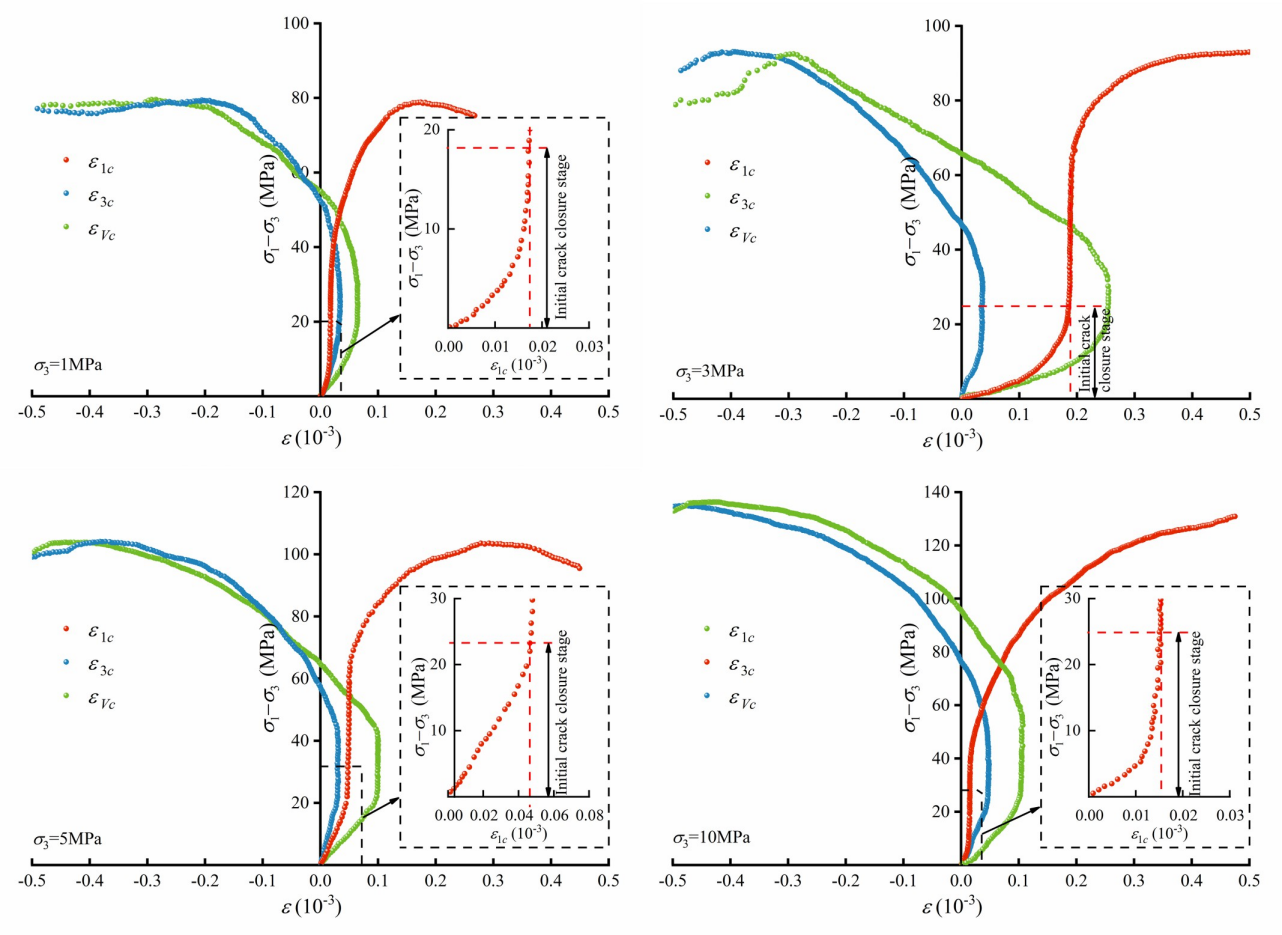
Crack strain and stress thresholds of micritic bioclastic limestone.

The deviatoric stress–crack strain curves of the micritic bioclastic limestone at confining pressures of 1 MPa, 3 MPa, 5 MPa and 10 MPa are represented in Fig 7. Because of the bioclastic content in the rock sample, the curves diverge slightly. The crack strain values in the initial crack closure stage differ among specimens. The deviatoric stress–crack strain curves of the rock under different confining pressures are similar, and all of them clearly show the initial crack closure stage, elastic deformation stage and crack propagation stage. Therefore, the rock crack evolution can be further explored to develop a crack-based constitutive model.

The stress–strength ratio *R_d_* is defined as the ratio of the deviatoric stress to the peak deviatoric stress, which characterizes the load bearing state of the rock. The dimensionless $R_{d}^{cc}$, $R_{d}^{ci}$, $R_{d}^{cd}$ and $R_{d}^{p}$ denote the ratios of deviatoric stress to peak deviatoric stress at *σ*_*cc*_, *σ*_*ci*_, *σ*_*cd*_ and *σ*_*p*_. The stress thresholds are shown in Table 4. The mean values of $R_{d}^{cc}$, $R_{d}^{ci}$ and $R_{d}^{cd}$ are approximately 0.23, 0.36 and 0.57, respectively.

**Table 4:** Stress thresholds, stress–strength ratio *R*_*d*_ and characteristic axial crack strain.

Confining pressure (MPa)	Crack closure			Crack initiation		Crack damage		Peak stress		
	*σ*_*cc*_ (MPa)	$R_{d}^{cc}$	$\varepsilon_{1c}^{cc}$ (10^−3^)	*σ*_*ci*_ (MPa)	$R_{d}^{ci}$	*σ*_*cd*_ (MPa)	$R_{d}^{cd}$	*σ*_*p*_ (MPa)	$R_{d}^{p}$	$\varepsilon_{1c}^{p}$ (10^−3^)
1	16.68	0.21	0.019	24.13	0.31	42.48	0.54	79.10	1	0.175
3	26.82	0.29	0.190	31.81	0.34	65.07	0.70	92.81	1	0.480
5	24.78	0.24	0.043	45.64	0.44	67.12	0.64	104.77	1	0.180
10	24.01	0.18	0.016	48.14	0.36	51.52	0.38	134.52	1	0.580

**Fig 8:**
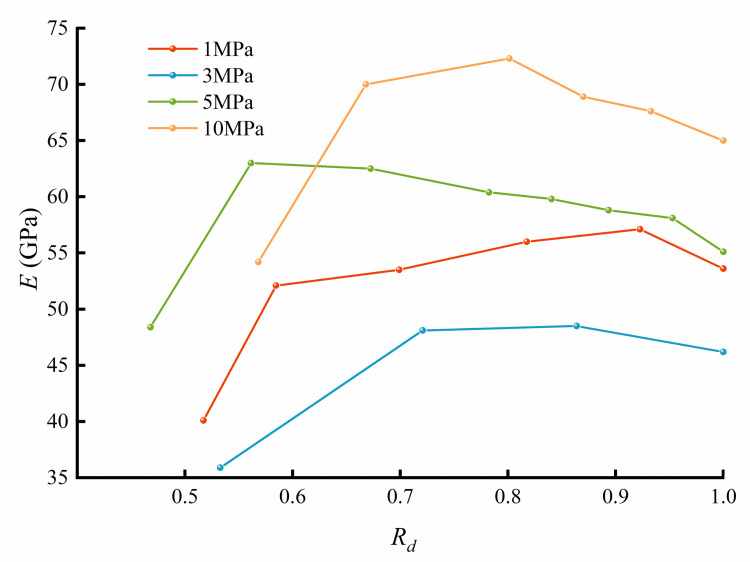
The rock elastic modulus results from the multilevel loading creep tests.

The elastic modulus of each rock specimen during each loading of the multilevel creep tests was obtained by the moving point regression technique and is plotted in Fig 8. The curves clearly show that the elastic modulus of the rock tends to increase and then decrease with the loading level. When the rock undergoes the first few stages of loading and creep, the rock microcracks and pores close, the rock weak structures are compressed, and the elastic modulus of the rock increases. As the loading and creep stages increase, new cracks accumulate, and the elastic modulus of the rock gradually decreases.

**Fig 9:**
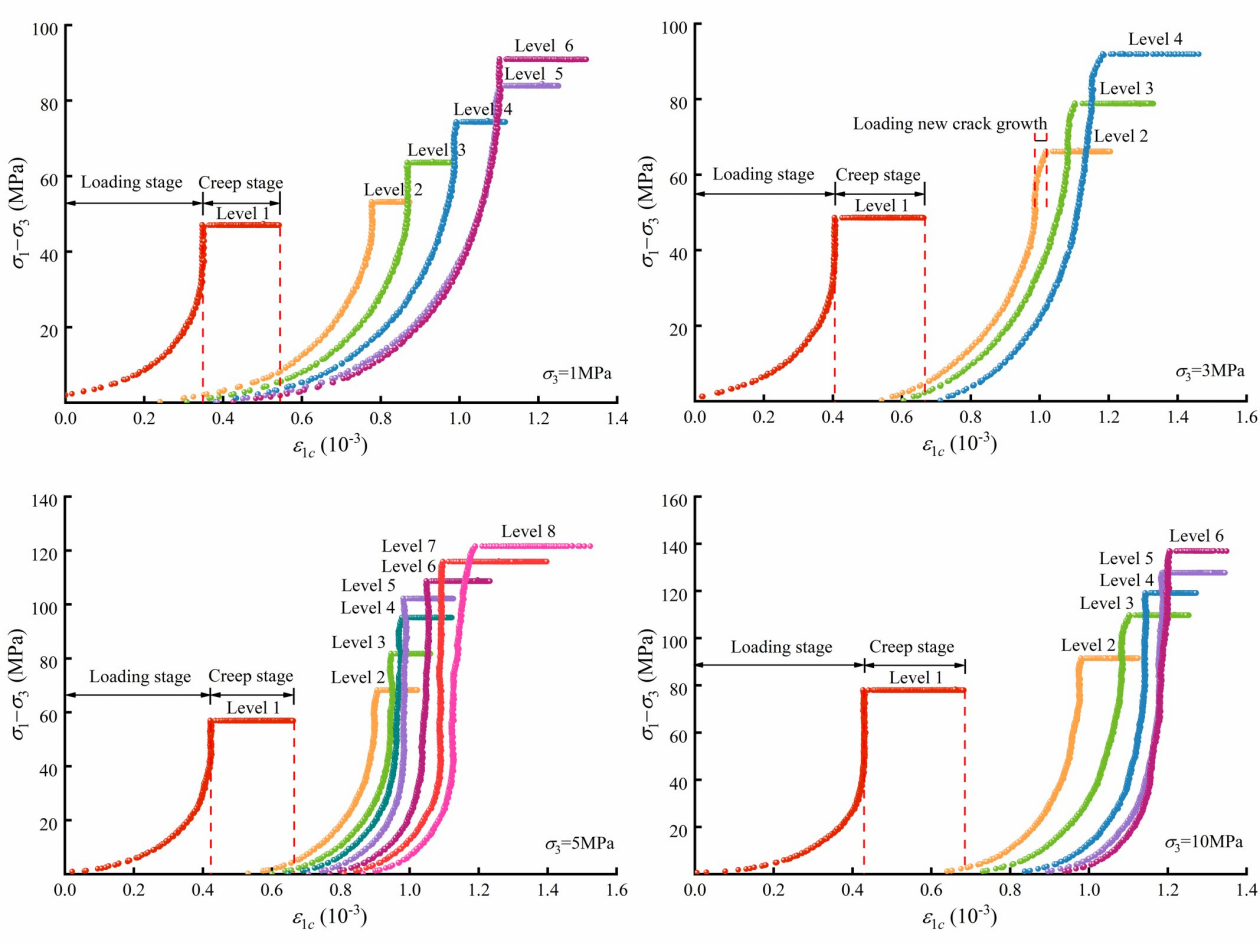
Axial crack strain results of micritic bioclastic limestone from triaxial compression creep tests.

According to the elastic modulus at each stage of loading, the axial crack strain curve of the rock loading and creep process can be obtained. As shown in Fig 9, the axial crack strain can be divided into a loading stage and creep stage. The crack evolution characteristics in the loading stage are similar to those of the triaxial compression test. The cracks in the rock gradually close as the deviatoric stress increases, and then the rock enters the elastic deformation state. As the axial loading increases, the axial crack strain deviates from a straight line, indicating that the deviatoric stress exceeds *σ*_*cd*_ and that axial cracks form in the rock, for example, see Fig 9(B). When the load holds at a constant deviatoric stress, the rock enters a creep state, in which the rock crack strain increases with holding time.

Taking level 1 of the 1 MPa confining pressure as an example, the percentage of creep crack strain is 64.8% of the initial crack closure strain, indicating that the creep crack strain at each creep level contributes negligibly to the total crack strain. Therefore, a constitutive model of rock that only considers transient rock deformation and ignores rock creep is inadequate for predicting the deformation and stability of underground caverns.

## 4. A unified transient creep constitutive model based on crack evolution

### 4.1 Crack strain element and elastic-crack constitutive model

To improve the constitutive model of rock, the transient crack evolution (loading) and the time-dependent crack evolution (creep) are unified based on crack strain. The following basic assumptions are introduced to study the time-dependent deformation characteristics of the micritic bioclastic limestone and establish the constitutive model.

(1) As proposed by Martin [36], the rock strain consists of elastic strain and crack strain.

(2) The elastic modulus of the rock matrix remains constant during a loading-creep-unloading process.

(3) The rock strain caused by loading is assumed to be independent of time and related only to the deviatoric stress level.

(4) As the elastic strain of the rock remains constant during creep, the creep strain is considered to be the time-dependent crack strain of the rock.

In triaxial compression tests, brittle rock failure is always related to crack evolution, according to a reference [44]. This means that the crack strain can characterize the mechanical response of the rock. Therefore, the characteristics of the crack strain evolution can be studied to establish a relationship between them, which can be used to predict the rock mechanical properties. Based on the characteristic stresses from the triaxial compression test, $R_{d}^{cc}$ is taken as 0.23, *and* $R_{d}^{ci}$ is taken as 0.36. As the initial crack varies to some extent from specimen to specimen, the initial crack closure strain $\varepsilon_{1c}^{cc}$ is normalized to the initial crack closure strain ratio *Rc* (Eq (5)) to obtain the initial crack closure law.


$$R_{c}^{}=\frac{\varepsilon_{1c}^{}}{\varepsilon_{1c}^{cc}}\;\left(0\leq R_{d}^{}\leq R_{d}^{cc}\right)$$ (5)

**Fig 10:**
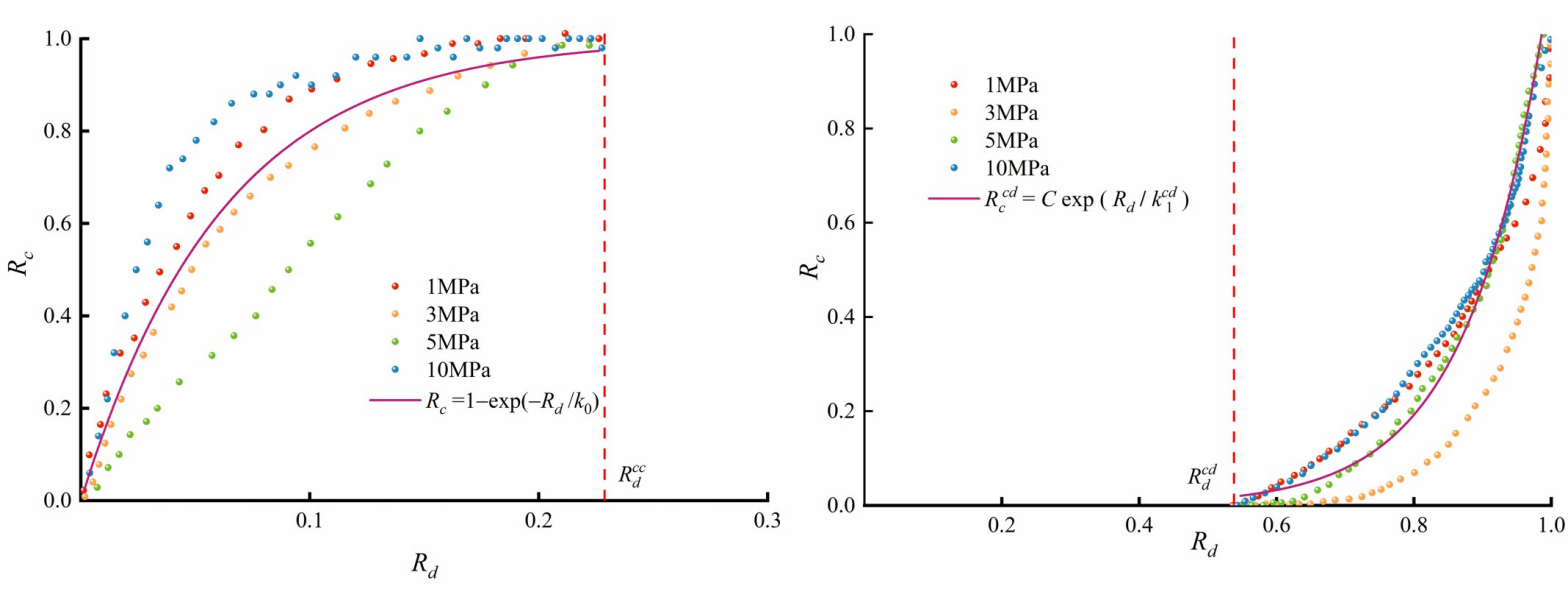
Axial crack strain evolution of micritic bioclastic limestone under triaxial compression tests: (a) initial crack closure stage and (b) new crack growth stage.

The axial initial crack closure strain ratios at *R*_*c*_ for confining pressures of 1, 3, 5 and 10 MPa are shown in Fig 10(A). It can be found that *R*_*c*_ shows the same evolutionary pattern for different confining pressures. The crack evolution at the initial crack closure stage can be accurately expressed by fitting Eq (6).

$$R_{c}^{}=1-e^{\frac{-R_{d}}{k_{0}}}\;\left(0\leq R_{d}^{}\leq R_{d}^{cc}\right)$$ (6)

where *k*_*0*_ is named the initial crack closure factor, which can be fitted for any test sample, reflecting the crack closure characteristics.

The crack strain appears in the axial direction of the rock when the load increases to $R_{d}^{cd}$, marking the beginning of the unstable crack growth stage. To study the axial crack evolution in this rock without considering the variability of the specimens, the axial crack strain is normalized by the ratio of axial crack strain *ε* 1*c* to peak axial crack strain $\varepsilon_{1c}^{p}$, or:

$$R_{c}^{}=\frac{\varepsilon_{1c}^{}}{\varepsilon_{1c}^{p}}\;\left(R_{d}^{cd}<R_{d}^{}\leq 1\right)$$ (7)

The curves for *R*_*c*_ versus *R_d_* are plotted in Fig 10(B). *R*_*c*_ begins to develop when the rock bearing state exceeds $R_{d}^{cd}$ and reaches 1, while *R_d_* equals 1. The evolutionary characteristics of the curves are the same for different confining pressures. The new axial crack strain evolution of the rock can be expressed by fitting Eq (8).

$$R_{c}^{}=Ce^{\frac{R_{d}}{k_{1}}}\;\left(R_{d}^{cd}<R_{d}^{}\leq 1\right)$$ (8)

where *k*_1_ denotes the axial new crack growth factor and *C* denotes the crack evolution factor.

**Fig 11:**
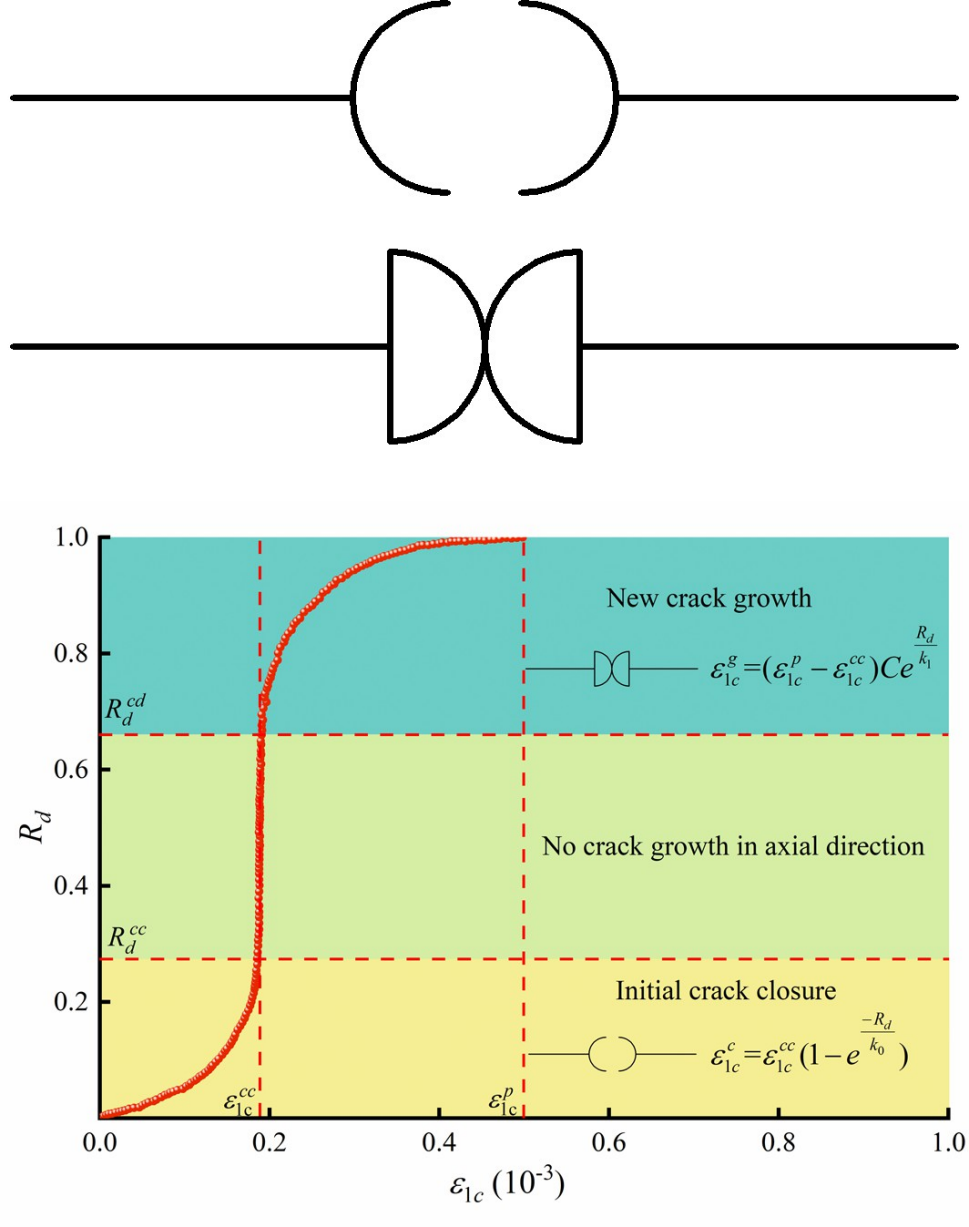
Rock crack evolution elements. (a) Initial crack closure element, (b) New crack growth element, (c) The role of axial crack strain elements.

To characterize the rock crack strain behaviour under compression, rock crack evolution elements are proposed, as shown in Fig 11. The element used to represent the transient initial crack closure of the rock is shown in Fig 11(A). Based on the crack evolution of the initial crack closure stage, the equation for the initial crack closure element can be expressed as:

$$\varepsilon_{1c}^{c}=\varepsilon_{1c}^{cc}R_{c}=\varepsilon_{1c}^{cc}(1-e^{\frac{-R_{d}}{k_{0}}})\;\left(0\leq R_{d}^{}\leq R_{d}^{cc}\right)$$ (9)

**Fig 12:**
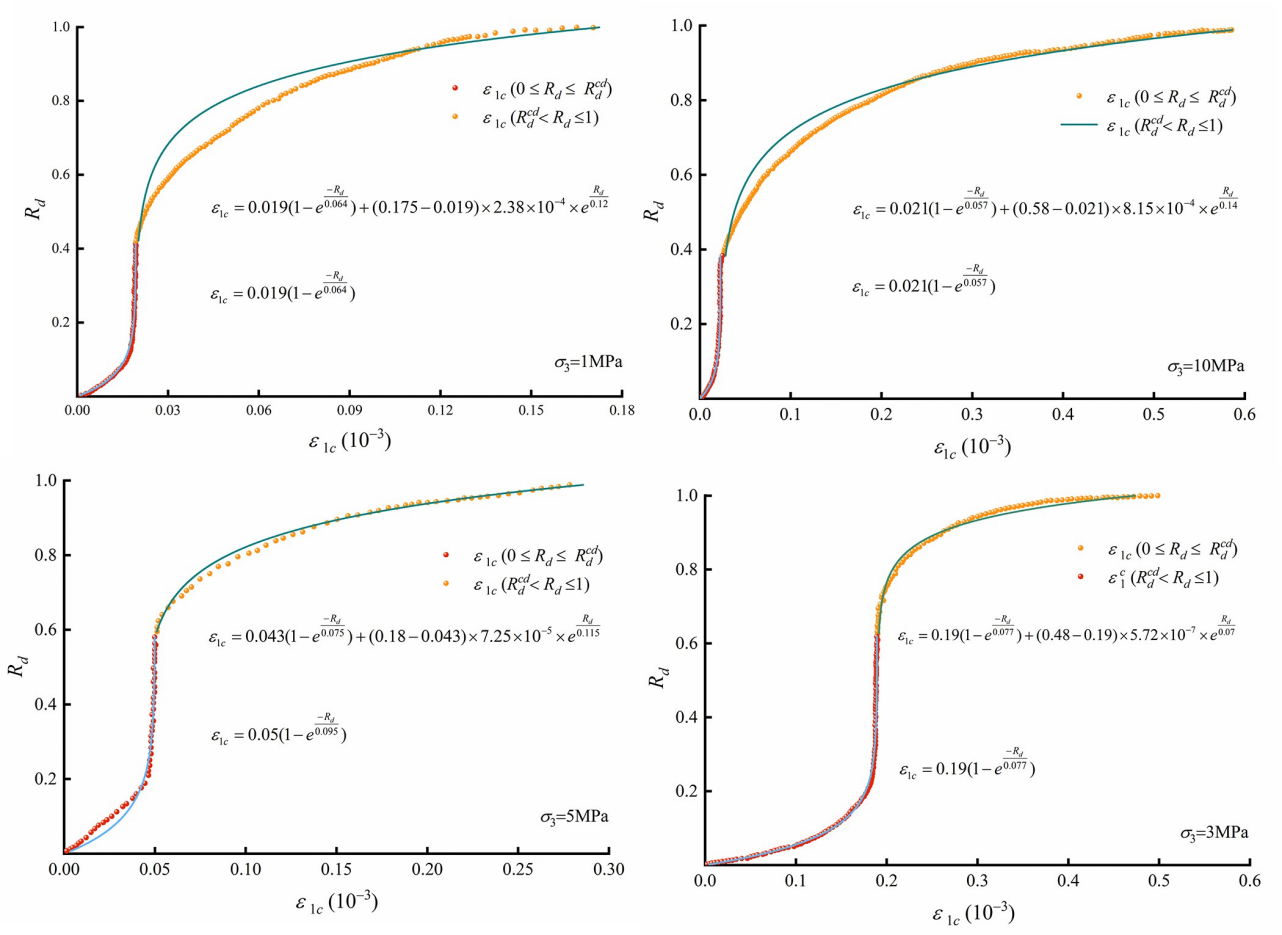
Axial crack strain results and model fitting from triaxial compression tests of micritic bioclastic limestone.

From Fig 11(C), when $R_{d}^{cc}$ ≤ *R*_*d*_ ≤ $R_{d}^{cd}$, no crack grows in the axial direction of the rock specimen. Thus, Eq (9) can also be used between $R_{d}^{cc}$ and $R_{d}^{cd}$. Similarly, the new crack growth element is shown in Fig 12(B), and the equation can be written as:

$$\varepsilon_{1c}^{g}=(\varepsilon_{1c}^{p}-\varepsilon_{1c}^{cc})R_{c}=(\varepsilon_{1c}^{p}-\varepsilon_{1c}^{cc})Ce^{\frac{R_{d}}{k_{1}}}\;\left(R_{d}^{cd}<R_{d}^{}\leq 1\right)$$ (10)

The role of the axial crack strain elements in the rock compression process can be seen according to Fig 11(C). The initial crack strain is represented by the initial crack closure element at the beginning of the rock loading until $R_{d}^{cc}$. While the rock bearing state is between $R_{d}^{cc}$ and $R_{d}^{cd}$, no new crack strain is generated in the axial direction. Once the rock bearing state exceeds $R_{d}^{cd}$, the new axial crack element comes into play, and the axial crack strain develops rapidly until *Rd* reaches 1.

The axial crack strain results from the triaxial compression tests can be represented by crack evolution elements. The crack strains $\varepsilon_{1c}^{cc}$ and $\varepsilon_{1c}^{p}$ are listed in Table 4. The LM (Levenberg‒Marquardt) algorithm was adopted to fit the axial crack strain data and obtain the element parameters (*C*, *k*_0_, *k*_1_). By fitting, the proposed initial crack closure and new crack growth elements can well characterize the evolution of the crack strain in the rock (Fig 12).

**Fig 13:**
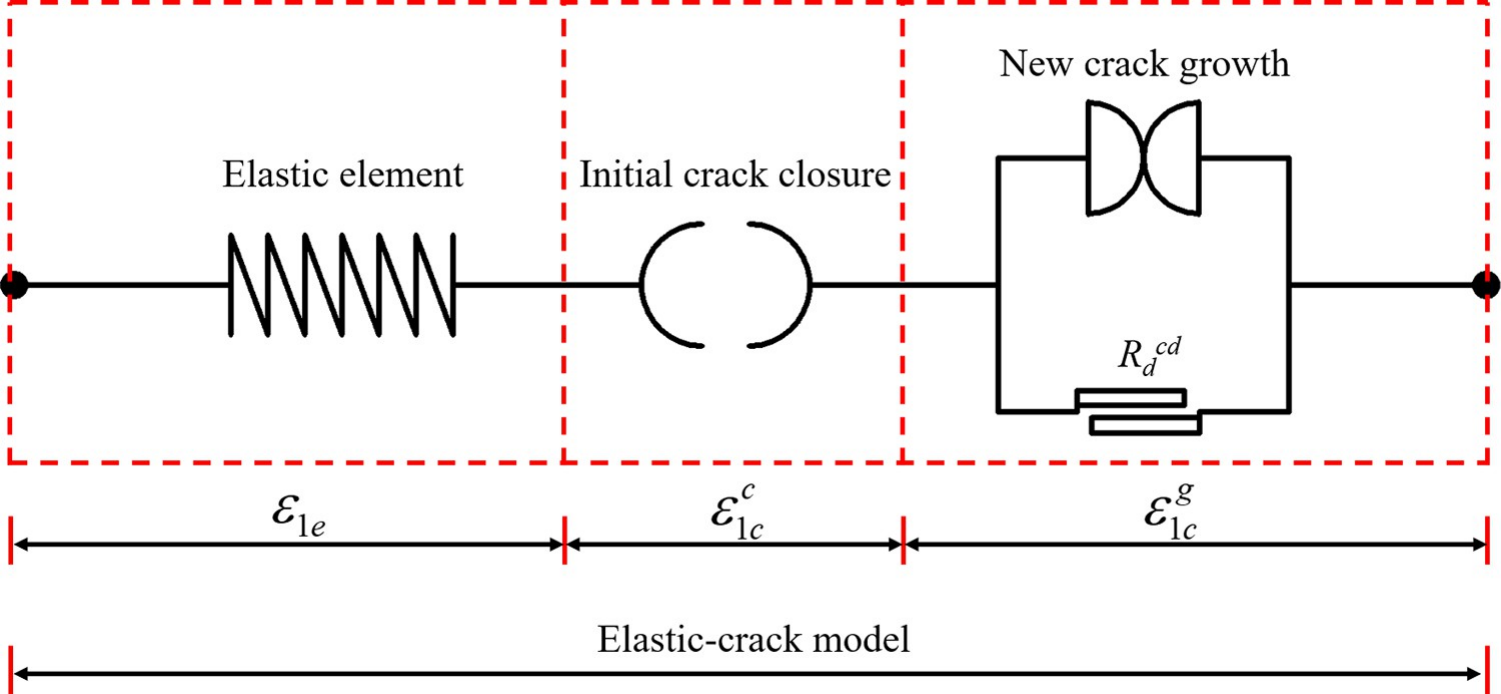
Elastic-crack model.

According to Eq (3), the total strain consists of elastic strain and crack strain. The elastic element is used to represent the elastic behaviour of the rock. Combined with the crack closure and new crack growth elements, a schematic representation of the elastic-crack constitutive model can be drawn as shown in Fig 13:

The elastic element follows Hooke’s law and can be expressed as Eq (11).


$$\varepsilon_{1e}^{}=\frac{R_{d}\sigma_{P}+\sigma_{3}(1-2\mu_{e})}{E}$$ (11)

Accordingly, the elastic-crack constitutive model can be expressed as:

$$\varepsilon_{1}^{}=\{\begin{array}{ll} \frac{R_{d}\sigma_{P}+\sigma_{3}(1-2\mu_{e})}{E}+\varepsilon_{1c}^{cc}(1-e^{\frac{-R_{d}}{k_{0}}}) & 0\leq R_{d}\leq R_{d}{}^{cd}\\ \frac{R_{d}\sigma_{P}+\sigma_{3}(1-2\mu_{e})}{E}+\varepsilon_{1c}^{cc}(1-e^{\frac{-R_{d}}{k_{0}}})+(\varepsilon_{1c}^{p}-\varepsilon_{1}^{cc})Ce^{\frac{R_{d}}{k_{1}}} & R_{d}{}^{cd}<R_{d}\leq 1 \end{array}$$ (12)

### 4.2 Creep crack strain element

**Fig 14:**
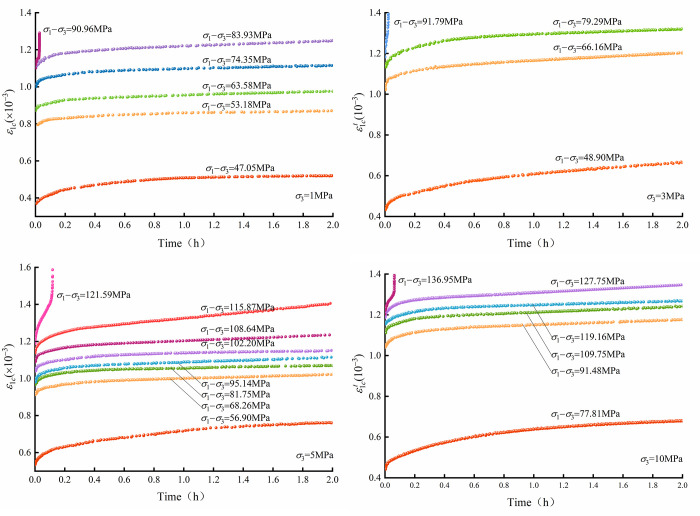
Axial creep crack strain results of micritic bioclastic limestone from triaxial multilevel creep tests with different confining pressures.

The creep crack strain of micritic bioclastic limestone is calculated by the crack strain equation in Eq (4). Axial creep crack strain–time curves are shown in Fig 14. The creep crack increment strain of the first level is larger than that of the later level. In addition, the curves show that there are noteworthy increases in crack strain between the first and second creep levels, indicating that the initial cracks are significantly adjusted in the first creep level. The typical characteristic of a creep crack strain curve is that the slope of the curve decreases with time until stabilizing. Furthermore, the steady-state slope of the creep crack strain curve increases with the loading level. As the loading level increases to a certain value, the rock crack strain rate first decreases, then stabilizes, and ultimately increases due to rock crack development.

**Fig 15:**
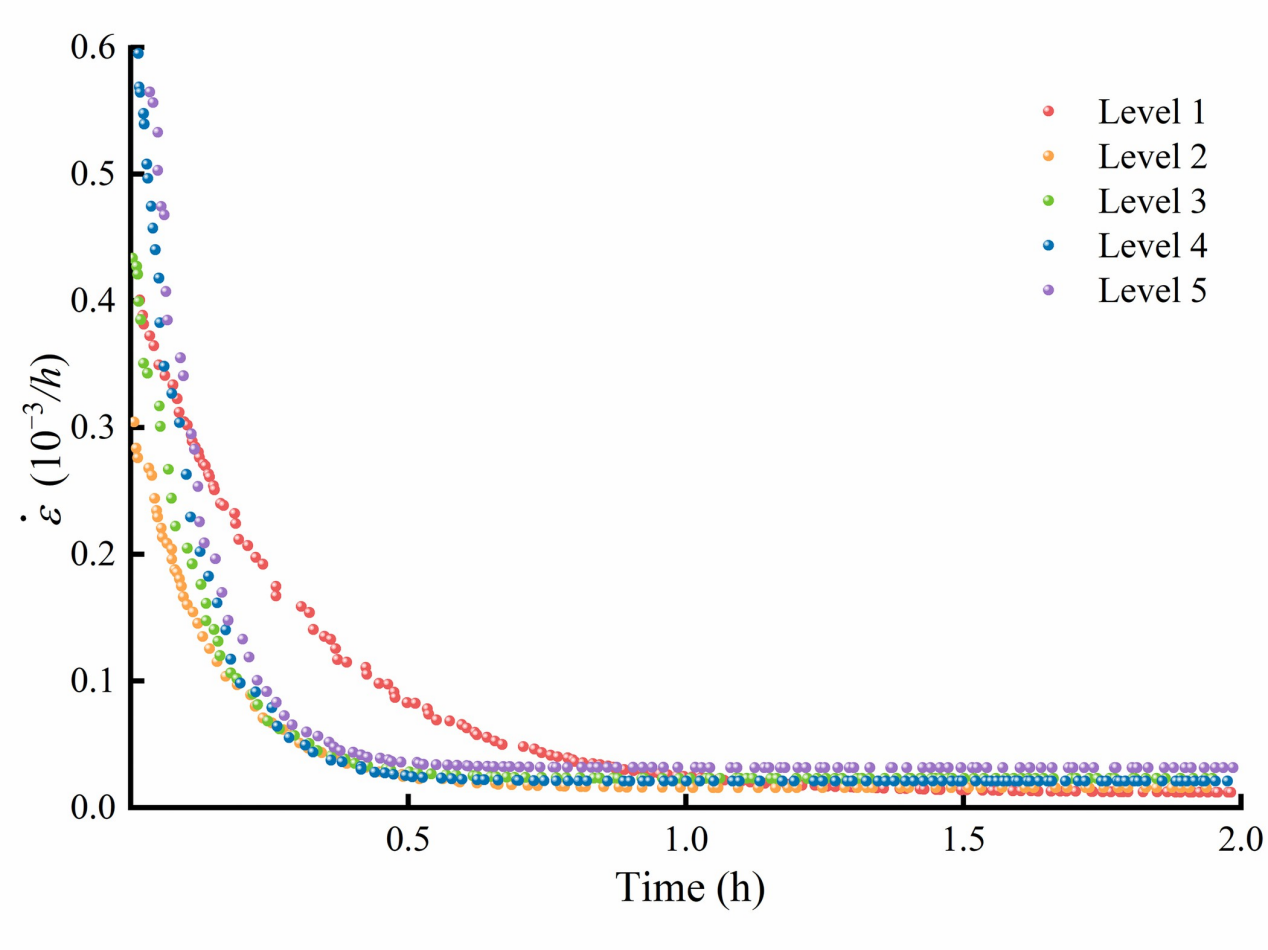
Axial creep crack strain rate of micritic bioclastic limestone from triaxial compression creep tests with 1 MPa confining pressure.

The axial creep crack strain rate of micritic bioclastic limestone under the triaxial multilevel compression creep test at a confining pressure of 1 MPa is plotted in Fig 15. The creep crack strain rate is large at the beginning of creep and gradually decreases and stabilizes with time. The higher the creep stress level is, the larger the steady creep crack strain rate. Studying the properties of the rock creep crack rate evolution will help to establish the rock creep crack evolution equation from the perspective of the rock failure mechanism. Therefore, it is recommended to focus on the crack growth velocity.

The Charles [46] law for subcritical crack extension in glass at a given stress state presents a power law expression for the microcrack growth velocity. The velocity of crack growth *V*_*c*_ is usually found to be uniquely related to the stress intensity factor (for a given set of environmental conditions). Other studies on rocks have found that the crack growth characteristics of rock are similar to those of glass [47]. According to the literature [48], some evidence indicates that there is a threshold below which no crack growth occurs. Considering the threshold effect, the crack growth velocity can be expressed as:

$$V_{c}=A<K_{i}-K_{0}{>}^{n}$$ (13)

To normalize the stress intensity factor difference, Eq (13) can be replaced by the following:

$$V_{c}=A{\left(\frac{<K_{i}-K_{0}>}{K_{c}-K_{0}}\right)}^{n}$$ (14)

where *V*_*c*_ denotes the crack growth velocity, *A* and *n* denote material parameters, *K*_0_ is the threshold of stress intensity factor for growth, *K*_*c*_ denotes the critical value of *K*_*i*_, and <> is the Macaulay bracket. The lack of an accurate initial crack length of the rock makes Eq (14) difficult to use directly because the stress intensity factor is related to the initial crack length. To overcome this difficulty, considering the similarity between microscopic cracks and macroscopic crack strains, the crack growth velocity *V*_*c*_ can be expressed by Eq (15) [11, 48].

$$V_{c}=A{\left(\frac{<\sigma_{i}-\sigma_{ci}>}{\sigma_{p}-\sigma_{ci}}\right)}^{n}$$ (15)

where *σ*_*p*_ denotes the peak deviatoric stress and *σ*_*ci*_ denotes the crack initial stress, which is the threshold of creep deformation.

*R*_*d*_, which characterizes the bearing state of the rock, is introduced to replace the stress in Eq (16) for dimensionless purposes. Considering that the crack growth velocity is proportional to the crack strain rate, the creep crack strain rate can be written as:

$${\dot{\varepsilon }}_{1c}=A{\left(\frac{<R_{d}-R_{d}^{ci}>}{1-R_{d}^{ci}}\right)}^{n}$$ (16)

Further simplification yields the expression for the steady-state creep crack strain rate:

$${\dot{\varepsilon }}_{1c}=A{\left(<R_{d}-R_{d}^{ci}>\right)}^{n}$$ (17)

Fig 15 shows that the axial creep crack strain rate of the rock gradually decreases with time and eventually stabilizes at the steady creep rate. The growth and adjustment of rock microcracks and pores when the rock is subjected to a constant load result in a gradual decrease in the rock crack growth velocity. The adjustment is expressed macroscopically as a decrease in the rock crack strain rate with time. Therefore, the rock creep crack rate evolution equation based on the steady state creep rate should be written as:

$${\dot{\varepsilon }}_{1c}=A{\left(<R_{d}-R_{d}^{ci}>\right)}^{n}+<R_{d}-R_{d}^{ci}>me^{-Bt}$$ (18)

where *m* and *B* denote two material parameters.

Taking an integral over Eq (18) gives:

$$\varepsilon_{1c}^{t}=At{\left(<R_{d}-R_{d}^{ci}>\right)}^{n}-\frac{m<R_{d}-R_{d}^{ci}>}{B}e^{-Bt}+F$$ (19)

where *F* denotes the parameter of loading crack strain and −*m*×($R_{d}^{ci}$−$R_{d}^{ci}$)/ *B+F* is the instantaneous loading crack strain.

**Fig 16:**
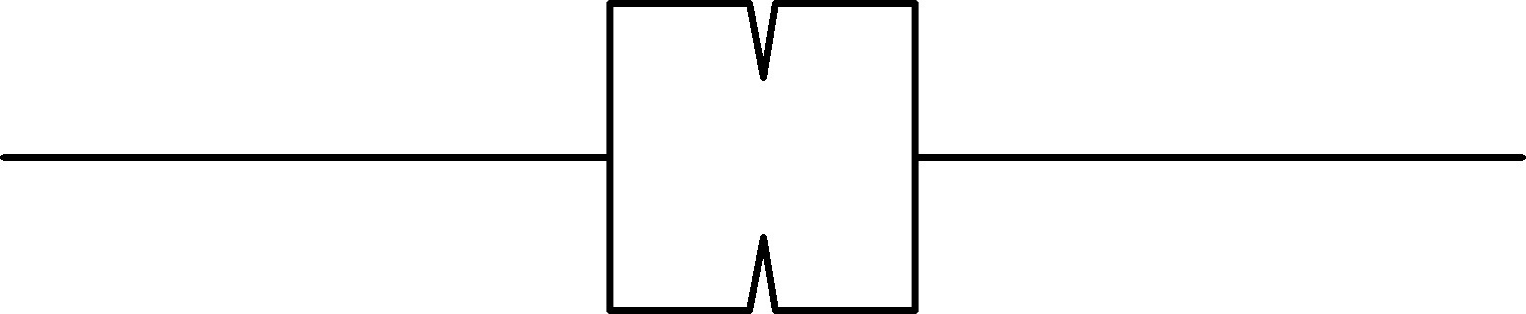
Rock creep crack evolution element (Mo’s element).

Previous researchers have proposed elements including springs, sliders, dashpots and fractional order elements that cannot characterise the evolution of rock crack strain over time. It is necessary to develop elements that characterise the evolution of rock creep crack. According to Eq 19, the element representing the creep crack strain of the rock, which is referred to as Mo’s element, is proposed, as shown in Fig 16.

**Fig 17:**
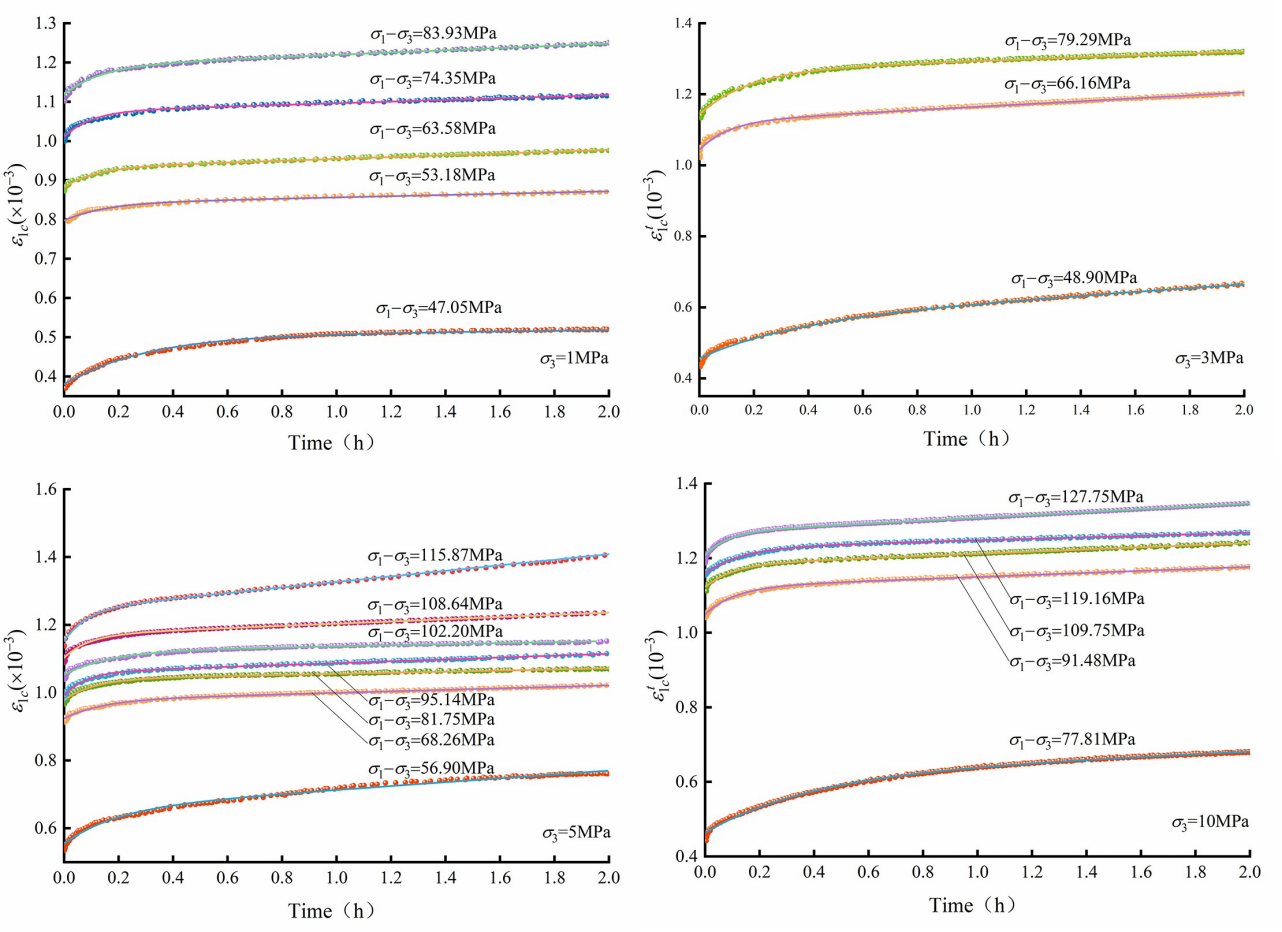
Axial creep crack strain results of micritic bioclastic limestone from triaxial multicreep tests and equation fitting. Note: line stands for fitting curve, and dots stand for test data.

Based on the creep crack strain evolution element proposed in this paper, the standard particle swarm optimization (SPSO) algorithm was employed to fit the experimental data. The parameters *A* and *n* are correlated, i.e., their values affect each other. To solve this problem, simplify the equation and reduce the number of parameters, the parameter *A* was set to 1. The fitting and test curves are shown in Fig 17, demonstrating that the creep crack strain element can describe the creep strain evolution of the rock well. The model parameters are shown in Table 5.

**Table 5:** Creep crack element parameters.

Confining pressure (MPa)	$R_{d}-R_{d}^{ci}$	*n*	*M*	*B*	*F*
1	0.147	2.321	2.783	3.469	0.498
	0.215	2.739	1.173	6.072	0.842
	0.329	3.391	1.330	8.870	0.932
	0.447	4.803	1.481	10.230	1.076
	0.553	5.830	1.458	10.686	1.187
3	0.162	1.561	2.273	3.752	0.551
	0.351	3.046	2.137	9.506	1.123
	0.494	5.136	1.375	6.803	1.266
5	0.098	2.642	3.079	1.556	0.761
	0.191	2.307	2.050	7.217	0.978
	0.302	3.458	1.879	9.209	1.040
	0.412	4.054	2.027	12.413	1.060
	0.471	6.361	0.677	4.228	1.132
	0.524	5.280	1.354	10.577	1.171
	0.583	4.651	1.888	11.842	1.246
10	0.198	2.115	1.998	2.574	0.618
	0.297	2.965	2.253	9.102	1.123
	0.431	4.209	1.381	9.826	1.183
	0.500	5.622	1.031	7.498	1.228
	0.560	5.788	1.209	11.118	1.270

**Fig 18:**
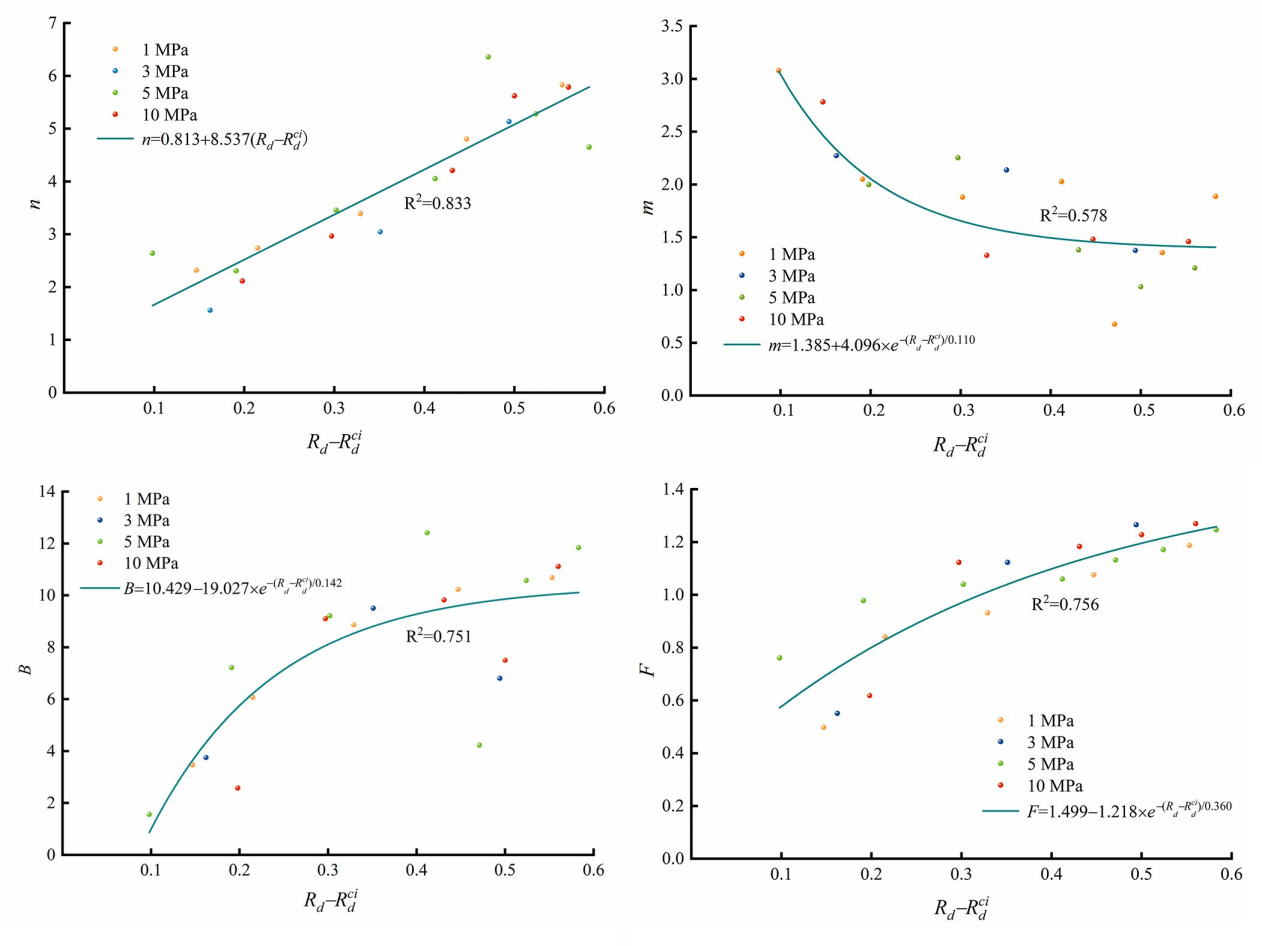
Creep crack element parameters.

The fitted creep crack strain element parameters *n*, *m*, *B* and *F* in Table 5 are plotted in Fig 18 under conditions of different confining pressures. The parameter *n* shows a positive linear relationship with *R_d_*, fitted as *n* = 0.813+8.537×($R_{d}-R_{d}^{ci}$). The parameter *m* shows an exponential relationship with *R_d_* and is fitted as *m* = 1.385+4.096×*e* ^−^ ($R_{d}-R_{d}^{ci}$)^/0.110^. The parameter *B* presents an exponential relationship with *R_d_* and is fitted as *B* = 10.429–19.027×*e* ^−^ ($R_{d}-R_{d}^{ci}$)^/0.142^. The parameter *F* also follows an exponential relationship with *R_d_* and is fitted as *F* = 1.499−1.218×*e* ^−^ ($R_{d}-R_{d}^{ci}$)^/0.360^. Notably, the parameters *n*, *m*, *B* and *F* are more related to *R_d_* than to the confining pressure. This also demonstrates the simplicity of the model proposed in this paper. The relationship between creep crack strain and bearing state through rock is revealed by the proposed model parameters.

### 4.3 Unified transient creep constitutive model (Mo’s model)

**Fig 19:**
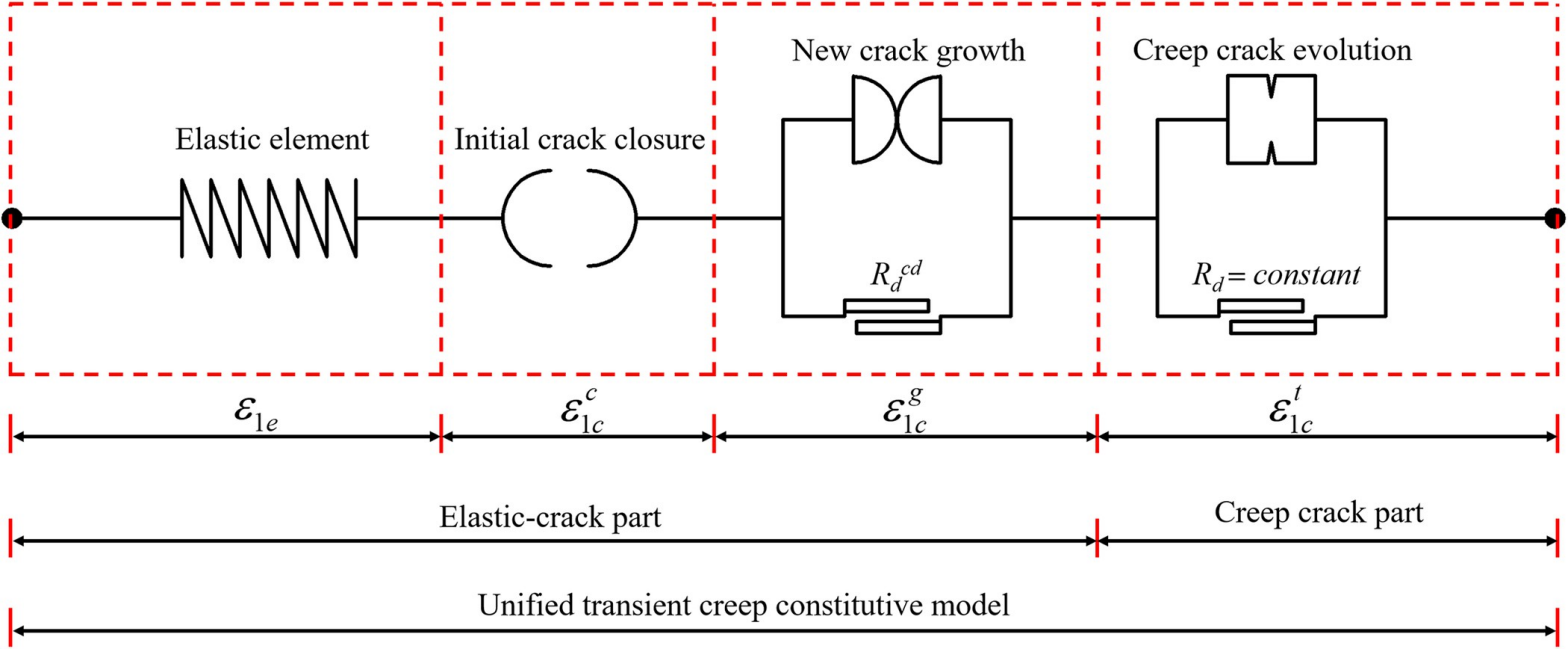
Unified transient creep constitutive model based on crack evolution.

Based on the above analysis of the transient and creep crack characteristics of the rock and the creation of crack elements, a unified transient creep constitutive model, which is referred to as Mo’s model, based on crack evolution under compression is developed, which includes an elastic element, an initial crack closure element, a new crack growth element, and a creep crack evolution element, as shown in Fig 19.

In accordance with the crack strain evolution characteristics in triaxial compression tests, combined with *R*_*d*_, the mechanical properties of the rock can be represented by the proposed constitutive model as:

$$\varepsilon_{1}=\{\begin{array}{ll} \varepsilon_{1e}^{}+\varepsilon_{1c}^{c} & 0\leq R_{d}\leq R_{d}{}^{ci}\\ \varepsilon_{1e}^{}+\varepsilon_{1c}^{c}+\varepsilon_{1c}^{t} & R_{d}{}^{ci}<R_{d}\leq R_{d}{}^{cd}\\ \varepsilon_{1e}^{}+\varepsilon_{1c}^{c}+\varepsilon_{1c}^{g}+\varepsilon_{1c}^{t} & R_{d}{}^{cd}<R_{d}\leq R_{d}{}^{p} \end{array}$$ (20)

Based on Eqs (12), (19) and (20), considering that the instantaneous loading crack strain equals −*m*×($R_{d}^{ci}$−$R_{d}^{ci}$)/ *B+F*, the rock transient creep constitutive model can be expressed as:

$$\varepsilon_{1}=\{\begin{array}{ll} \frac{R_{d}\sigma_{P}+\sigma_{3}(1-2\mu_{e})}{E}+\varepsilon_{1c}^{cc}(1-e^{\frac{-R_{d}}{k_{0}}}) & 0\leq R_{d}\leq R_{d}^{ci}\\ \frac{R_{d}\sigma_{P}+\sigma_{3}(1-2\mu_{e})}{E}+\varepsilon_{1c}^{cc}(1-e^{\frac{-R_{d}}{k_{0}}})+{\left(<R_{d}-R_{d}^{ci}>\right)}^{n}t-\frac{m<R_{d}-R_{d}^{ci}>}{B}(1+e^{-Bt}) & R_{d}{}^{ci}<R_{d}\leq R_{d}^{cd}\\ \frac{R_{d}\sigma_{P}+\sigma_{3}(1-2\mu_{e})}{E}+\varepsilon_{1c}^{cc}(1-e^{\frac{-R_{d}}{k_{0}}})+(\varepsilon_{1c}^{p}-\varepsilon_{1}^{cc})Ce^{\frac{R_{d}}{k_{1}}}+{\left(<R_{d}-R_{d}^{ci}>\right)}^{n}t-\frac{m<R_{d}-R_{d}^{ci}>}{B}(1+e^{-Bt}) & R_{d}{}^{cd}<R_{d}\leq R_{d}{}^{p} \end{array}$$ (21)

### 4.4 Verification of the elastic-crack model and unified transient creep constitutive model

**Fig 20:**
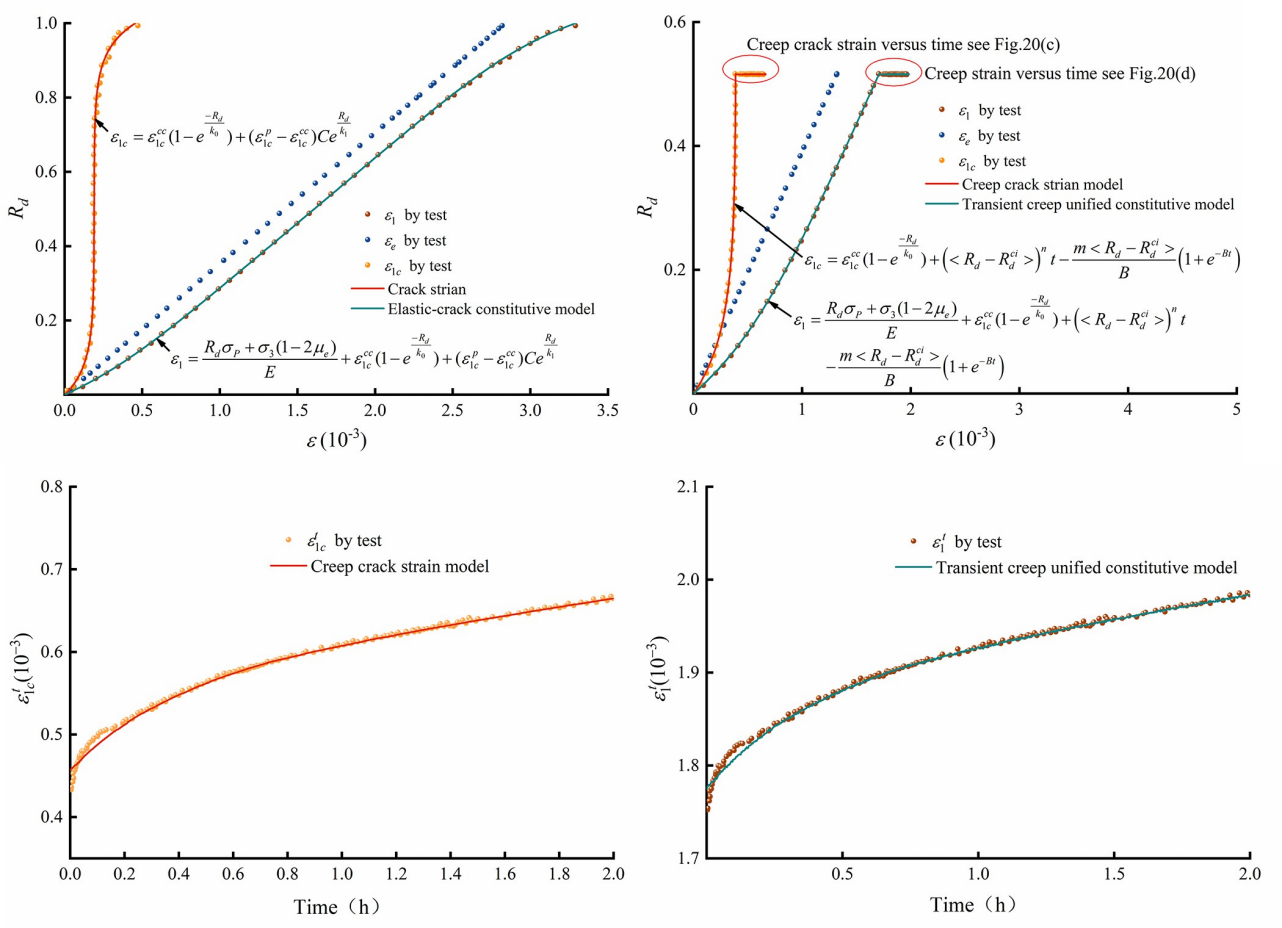
Application of the proposed constitutive models: (a) elastic-crack model, (b) transient creep unified constitutive model, (c) creep crack strain versus time and (d) creep strain versus time.

Based on the nature of rock failure, the elastic-crack model and the transient creep unified constitutive model are proposed in 4.1 and 4.3, respectively. To verify the feasibility of the proposed models, data from triaxial compression tests and first-level creep tests of micritic bioclastic limestone are used. The curves of *R*_*d*_ versus prepeak axial strain (crack strain, elastic strain, total strain) at 3 MPa confining pressure are presented in Fig 20(A). The fitting results show that the proposed crack strain elements and elastic-crack model can represent the axial crack evolution and transient mechanical response well. The transient creep unified constitutive model is applied to the first level of the 3 MPa confining pressure creep test of micritic bioclastic limestone. The fitting results show that the model can accurately represent both transient (Fig 20(B)) and time-dependent (Fig 20(C) and 20(D)) mechanical properties of the rock.

## 5. Conclusions

The crack strain evolution characteristics of micritic bioclastic limestone obtained from the No. 1 drainage tunnel of the Artashi Hydropower Project in Xinjiang, China, were studied by performing triaxial multilevel loading creep tests under different confining pressures. The following conclusions were obtained.

1. For the rock volumetric strain curve without a typical reversal arc to determine *σ*_*cd*_, an improved method is proposed based on the axial and lateral crack strain, which can determine the appropriate stress threshold for micritic bioclastic limestone.
2. *R*_*d*_, representing the bearing state of the rock, is introduced to investigate the crack evolution characteristics of the rock in triaxial compression tests. An elastic-crack model is proposed based on *R*_*d*_, and the crack evolution law, test data and model solution fit well with each other, indicating the suitability of this model to describe and predict the transient crack evolution of micritic bioclastic limestone.
3. Although the micritic bioclastic limestone has a high elastic modulus, the high content of bioclast matter makes the creep of the rock significant, which results in long-term deformation and fracture development throughout the life of engineering projects. From creep tests under different confining pressures, a creep crack strain evolution equation was obtained via the integral of the crack evolution rate and fits the experimental data closely.
4. Combining the crack closure, crack growth and creep crack elements, a unified transient creep constitutive model (Mo’s model) is proposed, in which the model parameters are related to *R*_*d*_. The verification shows that the model can accurately represent both transient and time-dependent mechanical properties of the micritic bioclastic limestone.

The new transient creep unified constitutive model (Mo’s model) can be further used in numerical programs to predict the surrounding rock deformation and to evaluate the stability and safety of the underground cavern of the Artashi Water Conservancy Project under different support designs and excavation schemes.
